# Construction of a prognostic model based on palmitoylation-related lncRNAs for assessing drug benefits in breast cancer

**DOI:** 10.3389/fimmu.2025.1656593

**Published:** 2025-10-27

**Authors:** Yan Wang, Mengsi Zhang, Yuqin Zhou, Zaozhuo Li, Xinglin Yi, Lin Ren, Yi Zhang

**Affiliations:** ^1^ Department of Breast and Thyroid Surgery, Southwest Hospital, Army Medical University, Chongqing, China; ^2^ Key laboratory of Chongqing Health Commission for Minimally Invasive and Precise Diagnosis and Treatment of Breast cancer, Chongqing, China; ^3^ Institute of Pathology and Southwest Cancer Centre, Southwest Hospital, Army Medical University, Chongqing, China; ^4^ Department of Information, Shanxi Provincial Armed Police Corps Hospital, Taiyuan, China; ^5^ Department of Respiratory and Critical Care Medicine, Southwest Hospital, Army Medical University, Chongqing, China

**Keywords:** palmitoylation-related lncRNA, breast cancer, prognostic model, potential therapeutic targets, drug benefits

## Abstract

**Background:**

The lncRNAs associated with protein palmitoylation in breast cancer (BC) remain largely unexplored.

**Methods:**

We retrieved transcriptome, proteome, and mutation data from TCGA-BRCA (BC), identified 592 palmitoylation-related lncRNAs (PRLs), constructed a prognostic model (PmPRLs) based on their characteristics. According to the score of the median risk, the “High-”and “Low” risk groups were distinguished. The predictive potential of PmPRLs for the prognosis of BC was determined through Kaplan-Meier (KM) survival analysis, ROC curve analysis, and risk scoring verification using the training set and validation set. The differences of PmPRLs in different risk groups were illustrated by using gene mutation frequency, immune function, tumour immune dysfunction and rejection (TIDE) score and drug sensitivity analysis. Based on this model, key feature LncRNAs were screened out. After the identified LncRNAs were verified by the external dataset TANRIC, a series of tumour phenotypic experiments were conducted to comprehensively demonstrate their role in tumourigenesis and development.

**Results:**

We identified 2 key feature lncRNAs, AC016394.2 and AC022150.4, as the most significant prognostic factors. Both of these lncRNAs exhibited high expression levels in the TCGA and TANRIC datasets and were closely associated with tumour cell growth, proliferation, and migration. More importantly, based on co-expression analysis, we proposed that AC016394.2 and AC022150.4 may respectively regulate SEC24C and ZNF611. Furthermore, these two lncRNAs enhanced the palmitoylation modification of these proteins.

**Conclusion:**

The insights regarding the potential roles of AC016394.2 and AC022150.4 can enhance our understanding of the mechanisms towards the pathogenesis and progression of BC.

## 1 Introduction

BC is a highly prevalent malignant neoplasm, and according to the recently published *Cancer Statistics 2025*, female patients constitute 32% of all newly diagnosed cases (1). Although advancements in treatment methods and early screening strategies have reduced the mortality, approximately 420,000 new cases are diagnosed annually, thereby imposing a significant health burden on families and society.

Protein palmitoylation (primarily S-palmitoylation) represents a novel lipid modification that relies on the coordinated actions of protein acyltransferases (PATs, specifically the ZDHHC family harbouring zinc finger Asp-His-Cys motifs) and protein acyl thioesterases (APTs). This process modulates protein function by altering their lipophilicity, thereby impacting tumour progression, drug response, and immune response (2). Such enzymes have been implicated in breast cancer, exhibiting diverse roles as either oncogenes or tumour suppressors and influencing various aspects of tumour biology. For instance, ZDHHC4 and ZDHHC22 expression are frequently silenced in breast cancer cell lines, and their functions appear to suppress tumour growth and metastasis by regulating RAS and mTOR/AKT (3–5). In contrast, ZDHHC3 palmitates B7-H4, an immune checkpoint, at Cys130 in breast cancer cells, thus preventing it from being degraded by lysosomes and maintaining B7-H4-mediated tumour immunosuppression (6). Beyond these examples, literature has also identified other ZDHHC PATs that directly or indirectly regulate breast cancer proliferation (7–9).

Long noncoding RNAs (lncRNAs) are non-coding RNA molecules which primarily influence the maintenance of proliferation signals, evasion of growth inhibitors, and resistance to cell death in tumour cells (10). Although lncRNAs are not proteins and do not undergo post-translational modifications, some reports suggest a correlation between lncRNAs and protein palmitoylation, in that lncRNAs co-expressed with PATs or APTs not only promote protein palmitoylation or depalmitoylation by affecting the interaction of their enzyme active sites, but are also associated with survival prognosis. Such as the lncRNA DUXAP8, which promotes the palmitoylation and then inhibits ferroptosis in hepatocellular carcinoma (11). Therefore, in-depth research on the association between lncRNAs and protein palmitoylation may facilitate the development of targeted therapeutics.

However, these lncRNAs, which we referred to as palmitoylation-related lncRNAs (PRLs), have been scarcely investigated to date in breast cancer. Therefore, the present study aimed to investigate whether PRLs could regulate BC malignant phenotypes. Furthermore, we sought to determine if a novel prognostic model based on these lncRNAs (PmPRLs) could accurately predict patient prognosis in BC. This research endeavours to provide new insights for both pharmacological interventions and clinical application in BC.

## 2 Materials and methods

### 2.1 Data retrieval and preparation of sample set

The data were retrieved from The Cancer Genome Atlas (TCGA; TCGA-BRCA cohort; https://tcga-data.nci.nih.gov/tcga/). The expression data (transcriptome data) include a total of 1,118 breast cancer (BC) tissue samples and 113 adjacent normal tissue samples, which are followed by 1,098 BC clinical data and 967 somatic mutation data. After differentiating the mRNAs from lncRNAs, the expression patterns of 30 genes related to palmitoylation (PALMs) reported in the literature were analysed in the BC tumour tissues and adjacent normal tissue samples, leading to the identification of 592 PRLs.

### 2.2 Development and validation of PmPRLs

After filtering, a total of 1,098 BC samples were randomly divided into a training set (n = 594) and a validation set (n = 594) at a 1:1 ratio. A total of 22 PRLs associated with the prognosis of BC were identified in the training set using univariate Cox regression analysis. A subset of these PRLs was selected by LASSO-Cox regression analysis to prevent overfitting. A predictive model for estimating the prognosis of BC was subsequently developed based on the training set, using multivariate Cox regression analysis. It was determined that the optimal number of lncRNAs corresponding to the lowest error point was *17*, where prognostic models were constructed utilising these specific lncRNAs from which model formulas were derived. The coefficients of 9 specific PRLs were incorporated into the model to enhance prognostic accuracy through screening. The coefficients of the 9 PRLs used for model development were as follows: AC096642.2 (0.506456858), AP000331.1 (-0.38367969), AC016394.2 (0.283605425), AC090510.2 (-0.553689916), AC011815.1 (-0.580334682), AC022150.4 (0.288210056), Z68871.1 (0.330530176), AL109936.9 (-0.711784776), and AL162386.2 (-0.641615803). The patients were categorised into “High-risk” or “Low-risk” groups based on the median risk score obtained using the model. The overall survival and survival rates across different clinical subgroups were compared by KM analysis. The predictive performance of the model was evaluated using ROC curves, and the area under the ROC curve (AUC) values were calculated at 1-, 2-, and 3-year intervals.

### 2.3 Classification of tumours based on PRL expression

The ConsensusClusterPlus package in R v4.4.2 was used to classify the 1,098 BC samples into three subtypes based on the expression levels of PRLs. Principal component analysis (PCA) was performed using the ggplot2 package in R to analyse the variations in the data. The differences in overall survival (OS) across the subtypes were examined by KM analysis. The ESTIMATE and CIBERSORT packages in R were used to quantify the immune cells and stromal cells in the BC samples.

### 2.4 Construction of nomogram and analysis of risk differences

The “rms package” in R was used to create a nomogram based on the clinical factors and risk scores. The nomogram predicted the survival probabilities at 1, 2, and 3 years based on the total score. The performance of the model in predicting patient survival was evaluated using calibration and ROC curves. The differentially expressed genes (DEGs) in the “High-risk”and “Low-risk” groups were identified after analysing the risk differences. PCA was performed using the “scatterplot3d package” in R to assess the differences in the PRLs used for model development between the two risk groups. The feature enrichment annotations and pathways of the DEGs in each group were identified by Gene Ontology (GO), Kyoto Encyclopedia of Genes and Genomes (KEGG), and Gene Set Enrichment Analysis (GSEA).

### 2.5 Integrated analysis of somatic mutations and drug sensitivity

The maftools package in R was used to identify the somatic mutations in patients with BC. The mutation spectra of the “High-risk” and “Low-risk” groups were compared using waterfall plots. The IC_50_ values of drugs commonly administered to patient groups with different types of BC were estimated using the oncoPredict algorithm to assess drug sensitivity and evaluate the potential therapeutic effects based on the drug sensitivity profiles.

### 2.6 Screening of differentially expressed lncRNAs selected for model development

The Limma, survival, and survminer packages in R were used to verify the expression, OS, and clinical grouping models of the 17 PRLs selected for model development. The AC016394.2 and AC022150.4 lncRNAs were selected as research objects, and the Tidyverse package in R was used to screen the genes co-expressed with AC016394.2 and AC022150.4.

### 2.7 Prediction of co-expressed genes at single-cell landscape

Gene set related to breast cancer was retrieved from http://tisch.compbio.cn/, and the BRCA_EMTAAB8107 gene set was finally selected for analysis. After annotating the cell clusters, the immune-related cells with the most significant differences in co- expressed genes among various immune cells were identified.

### 2.8 Cell culture

The luminal-type MCF-7, Her-2-type SKBR3, BT474 and TNBC-type MDA-MB-231, MDA-MB-468, and SUM-159 human BC cell lines, as well as normal breast epithelial MCF-10A cells, were purchased from the American Type Culture Collection (ATCC). All the cells were cultured in Dulbecco’s Modified Eagle Medium (DMEM) supplemented with high glucose (Gibco, USA) and 10% foetal bovine serum (Gibco, USA) at 37°C in a humidified incubator with 5% CO_2_.

### 2.9 Fluorescence-based real-time quantitative polymerase chain reaction

The total RNA was extracted as protocol, and 2 × ChamQ Universal SYBR qPCR Master Mix (Q711-02, Vazyme, Nanjing, China) were used for qRT-PCR. The following primer sequences used for qRT-PCR (details in **Table 1**).

**Table 1:** Real-time RT-qPCR primer sequences.

Species	Molecule	Forward (5’-3’)	Reverse (5’-3’)
Human	AC016394.2	ACCCGAAGGAAGACTCCTCT	ATCCTGGTGTGCAGAATGGG
Human	AC022150.4	GATCTGTGTGGACCCCAGAA	CTCCGGTCCCCAGCATAGAA
Human	SEC24C	CGTCTCCTACAATGCCATCAGG	GGTGACAAAGCCAACGCGGATT
Human	ZNF611	CCCTTCACAGAGGGCTTTGTAC	GCAATGTCCCTGTGTGGATCAC
Human	GAPDH	CCATGGGTCGAATCATATTGGA	TCAACGGATTTGGTCGTATTGG

### 2.10 Transfection

The overexpression and siRNA plasmids of AC016394.2 and AC022150.4 were purchased from Qingke Biotechnology Co., Ltd., Wuhan, China. The cells were seeded into 6-well plates at 1×10^5^, and transfection was performed after 24h using Lipofectamine 3000, according to the standard protocol.

### 2.11 Assessment of cell proliferation potential

Cell viability was assessed using CCK-8 assays with a HY-K0301 kit (Shanghai, China), and 1000 cells were seeded and incubated for 2 weeks for the colony formation assay. The cells were fixed with paraformaldehyde for 0.5 h and stained with crystal violet by incubating for 1 h, following which the colonies were enumerated and images were captured at 24, 48, 72, and 96 h.

### 2.12 Cell scratch assay

The cells were inoculated into a 6-well plate, and the tip of a sterile 200μL pipette was used to create scratches on the surface of the culture when the cells reached a confluence of 90%. The detached cells were subsequently washed with PBS, following which serum-free medium was added to the culture, and the cells were incubated for 24 h. Images of the cell plates were captured using an inverted microscope at 0 and 48 h. The pixel area of the scratch was calculated using Image J software, and the scratch recovery rate was calculated as: (pixel area at 0 h - pixel area at 48 h)/pixel area at 0 h.

### 2.13 Mouse xenograft model and immunohistochemistry (IHC) analysis

BALB/c nude mice, aged 3–4 weeks, were purchased from Southern Model Biotechnology Co., Ltd. (Shanghai, China). The mice were divided into a control group 1, AC016394.2 overexpression group, control group 2, and AC092894.1 overexpression group. Approximately 3 × 10^6^ SUM159/BT-474/MCF-7 vector or SUM159 BT-474/MCF-7-AC022150.4 cells were implanted *in situ* onto the right adipose pads of the mice. The mice were euthanised after 14 days and the tumours were surgically removed.

The tumour tissues were embedded in paraffin, sectioned, deparaffinised, and rehydrated through a graded alcohol series. Antigen retrieval was subsequently performed with Tris-EDTA buffer, followed by incubation with 3% H_2_O_2_ for 30 min to block the activity of endogenous peroxidases. The slides were blocked with 10% normal goat serum (BOSTER Biological Technology co.Ltd.) for 30 min at 37°C, and subsequently incubated overnight at 4°C with mouse anti-human Ki-67 antibody (cat. no. ZM-0166; ZSGB-BIO, Beijing, China). The sections were rinsed with wash buffer and subsequently incubated with an anti-mouse/rabbit polymer kit (EnVision Plus; Dake) for 30 min at 20°C. The IHC samples were incubated with a secondary antibody for 30 min at 20°C the following day, followed by detection with 3,3’-Diaminobenzidine (DAB). The stained sections were finally observed under a microscope, and images were captured.

### 2.14 ABE assay

Protein palmitoylation modification was detected using the ABE method. The HA-SEC24C/HA-ZNF611 plasmid was transiently transfected into HEK293T cells, which were then collected and washed with cold PBS after 48 hours. Subsequently, sh-LncRNA (sh-AC016394.2/AC022150.4) was transfected 24 hours prior to cell collection. The cells were lysed on ice for 1 hour in lysis buffer containing 50 mM NEM (Sigma-Aldrich, E3876) and a protease inhibitor. The target protein was immunoprecipitated using an anti-HA antibody (Beyotime, AF0039) and protein A/G magnetic beads (Beyotime, P2108-5ml). The purified protein was incubated at room temperature with a buffer containing 1 M HAM (Sigma-Aldrich, 814441) for 1 hour, followed by treatment with a buffer containing 5 μM biotin-BMCC (Invitrogen, 21900) at pH 6.2 for an additional hour at 4°C. Finally, the samples were analysed via Western blotting using an anti-HA antibody (1:1000; Beyotime) and HRP-streptavidin (1:5000; Beyotime).

### 2.15 Western blot

The cells were washed three times with PBS and subsequently lysed on ice for 15 minutes using RIPA buffer (Beyotime, P0013B) supplemented with PhosSTOP™ protease inhibitor (Roche, 04906837001). Following lysis, the samples were centrifuged at 15,000 rpm for 15 minutes to obtain the supernatant. After quantifying the protein concentration via BCA assay, an equal amount of protein was subjected to SDS-PAGE. The specified antibody was employed as a probe, and detection was performed utilising a chemiluminescence substrate (Pierce). The antibodies used in this study included: anti-SEC24C (Novus, NBP2-94294-0.02ml) for human SEC24C; anti-ZNF611 (CUSABIO, CSB-PA026891GA01HU) for human ZNF611; anti-Ki67 (Proteintech, 28074-1-AP) for mouse Ki67; anti-β-actin (GenScript, A00702); anti-GAPDH (Proteintech, 60004-1-Ig); HRP-linked anti-Mouse IgG (CST, 7076); and HRP-linked anti-Rabbit IgG (CST, 7074).

### 2.16 Statistical analyses

The data are presented as the mean ± standard deviation (SD), and the statistical analyses were performed using R v4.4.2 (https://www.r-project.org/) and SPSS v25.0. Statistical significance was considered at *P* < 0.05 and indicated by asterisks: *P< 0.05, **P < 0.01, ***P < 0.005, and ****P < 0.001.

## 3 Results

### 3.1 Schematic representation of PRLs and research methodology

**Figure 1:**
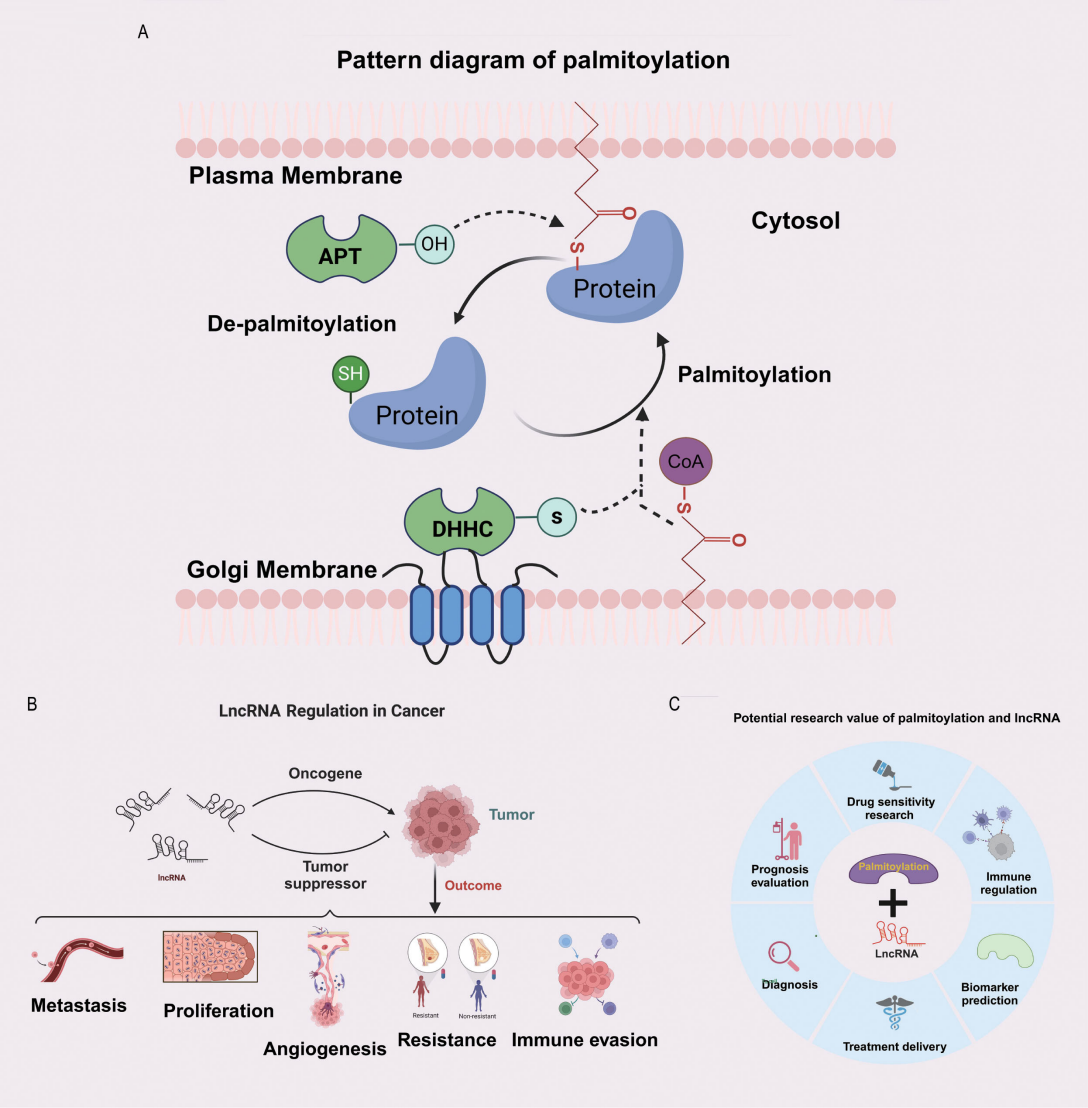
Potential significance of the combined study of palmitoylation and LncRNA. **(A)** The diagram of protein palmitoylation. **(B)** The bidirectional regulatory role of LncRNA in tumours. **(C)** The potential research value of palmitoylation and LncRNA.

The process of protein palmitoylation is illustrated in **Figure 1A**. LncRNAs play critical roles in promoting or suppressing the development of cancer, and several aspects of lncRNA biology, including their functions in tumour metastasis, tumour proliferation, angiogenesis, drug resistance, and immune escape, have been extensively investigated (**Figure 1B**). Protein palmitoylation and lncRNAs have substantial research potential, as depicted in **Figure 1C**. The research methodology is depicted in a flowchart in **Supplementary Figure S1**.

### 3.2 Determination of expression of PRLs and model development

**Figure 2:**
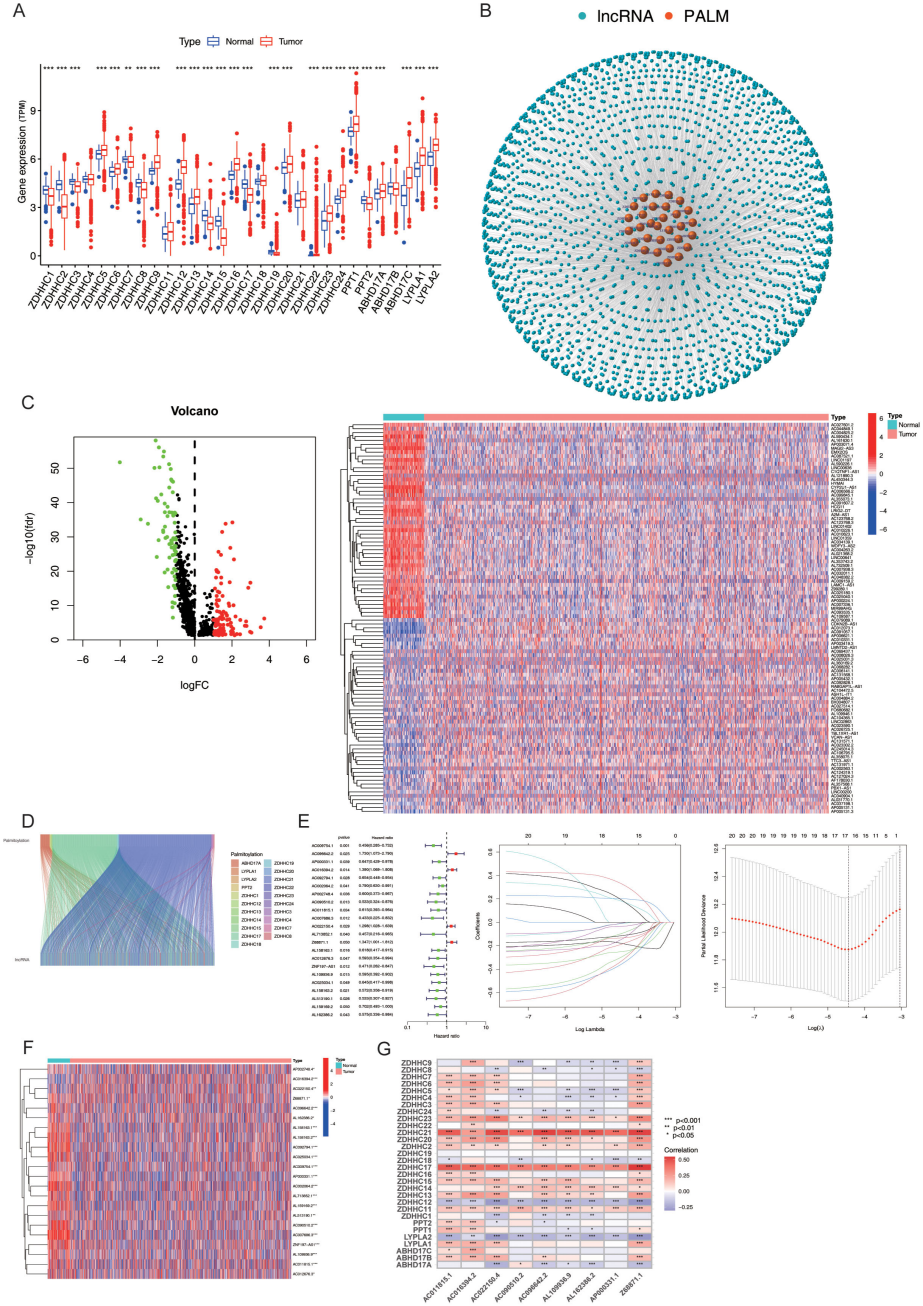
Expression and Prognostic Model of PRLs. **(A)** Differential expression analysis of 30 palmitoylation-related genes (PALMs) in breast cancer cells. **(B)** Node diagram illustrating the co-expression relationship network between PALMs and LncRNA. **(C)** Analysis of differential expression of LncRNA in breast cancer cells. **(D)** Sankey plot depicting the correlation between differentially expressed lncRNAs and PALMs. **(E)** Distinction between the training set and test set for “high-risk” and “low-risk” groups, including a forest plot of 22 lncRNAs associated with prognosis, as well as results from Lasso regression analysis. **(F)** Heat map representing the differential expression of 22 lncRNAs in breast cancer cells. **(G)** Correspondence between the nine PRLs utilised for model development and PALMs. *P < 0.05, **P<0. 01, ***P<0. 001.

The transcriptome expression data of 1,118 BC tissue samples and 113 adjacent normal tissue samples were retrieved from TCGA. The expression levels of 30 PALMs reported in the literature (**Supplementary Table S1**) were determined after distinguishing the mRNAs from lncRNAs, and their differential expression between tumour tissues and adjacent normal tissue samples was analysed (**Figure 2A**). The lncRNAs that were co-expressed with PALMs were identified, and the network nodes were subsequently visualised (**Figure 2B**). The nodes were then statistically analysed to identify the lncRNAs that were differentially expressed between tumour tissues and adjacent normal tissue samples (**Supplementary Table S2**). The differentially expressed lncRNAs were represented using volcano plots and heat maps (**Figure 2C**). The correlation between the PALMs and differentially expressed lncRNAs was illustrated using a Sankey diagram (**Figure 2D**), and a total of 592 PRLs were finally identified (**Supplementary Table S3**). A total of 1,098 clinical samples were retrieved from TCGA-BRCA, and the lncRNA expression data were combined with the survival data to identify 22 lncRNAs associated with the prognosis of BC (**Figure 2E**). In our analysis, we aimed to maximise sample matching by merging expression and survival datasets. Ultimately, we identified a total of 1,098 cases and divided them into training and validation sets at a ratio of 1:1 (n_1_=n_2_=594). The training set was used to construct a prognostic model, and the accuracy of the model was validated using the test set. The model was represented using the following formula, which was used to calculate the risk score for each sample: *Risk score=* $\sum_{n=1}^{\infty }\left(e_{n}\times \beta_{n}\right)$ .As aforementioned, the samples were divided into the Risk-high and Risk-low groups, based on the median risk score. The results of univariate analysis revealed that 22 lncRNAs were associated with the prognosis of BC, of which 17 characteristic lncRNAs were identified by Lasso regression analysis (**Figure 2F**). Statistical analysis of the clinical data revealed no significant differences in the clinical characteristics between the training and test sets (*P* > 0.05; **Supplementary Table S4**). The correlation between the PALMs and the 9 PRLs used for model development was represented using a heat map to observe the corresponding relationships between these PRLs and PALMs (**Figure 2G**).

### 3.3 Validation of the survival prediction potential of PmPRLs

**Figure 3:**
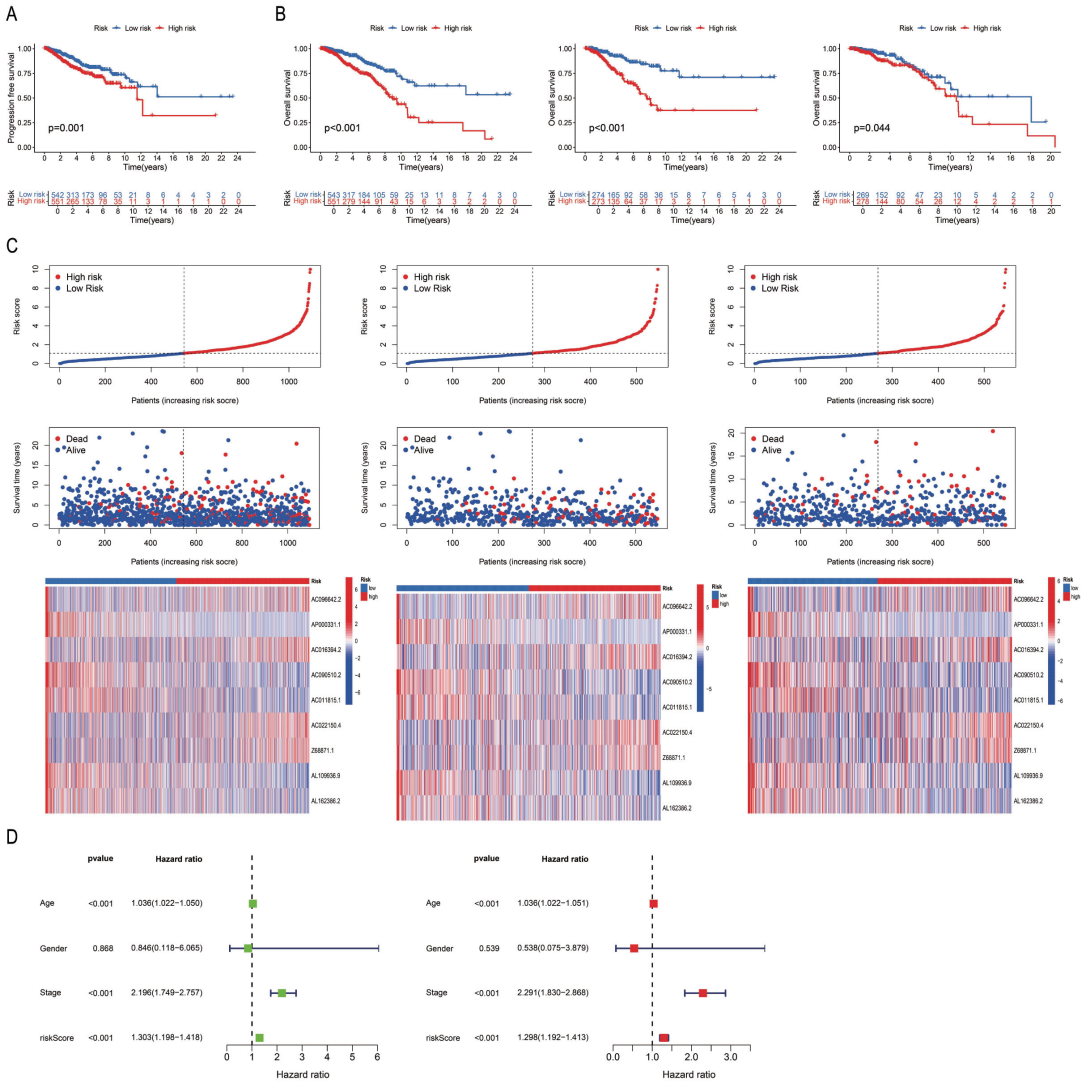
Validation of the correlation between PmPRLs and survival rate in “high-risk” and “low-risk” groups. **(A)** Progression-free-survival (PFS) in two groups. **(B)** Overall Survival (OS)of patients in the sample, training, and test sets. **(C)** Risk scores and survival status. **(D)** Correlation between model prediction and prognostic outcomes.

The differences between the “High-risk” and “Low-risk” patient groups were analysed using the survival and survminer packages in R. The findings revealed significant differences in progression-free survival (PFS; *P* < 0.05; **Figure 3A**) and OS rates (*P* < 0.05; **Figure 3B**) among the sample, training, and test sets derived from the “High-risk” and “Low-risk” groups. The mortality rate increased with higher risk scores across all three groups. The risk score, survival status, and results of differential heat map analysis are discussed hereafter. Among the 9 PRLs selected for model development in this study, AC096642.2, AC016394.2, AC022150.4, and Z68871.1 were identified as high-risk lncRNAs (associated with poor prognosis), while AP000331.1, AC090510.2, AC011815.1, AL109936.9, and AL162386.2 were low-risk lncRNAs (associated with good prognosis), and these classifications remained consistent across the sample, training, and test sets (**Figure 3C**). Further classification of the tumour samples indicated that the PRL-based model developed herein exhibited significant potential for the classification of BC tumours and risk stratification (**Supplementary Figure S2**). Due to variations in age, stage, and risk score (*P* < 0.05), the accuracy of PmPRLs can be attributed to the inclusion of PRLs, which serve as an independent prognostic factor of BC (**Figure 3D**).

### 3.4 Association between PmPRLs and clinical traits, and functional analysis of “High-risk” and “Low-risk” patient groups

**Figure 4:**
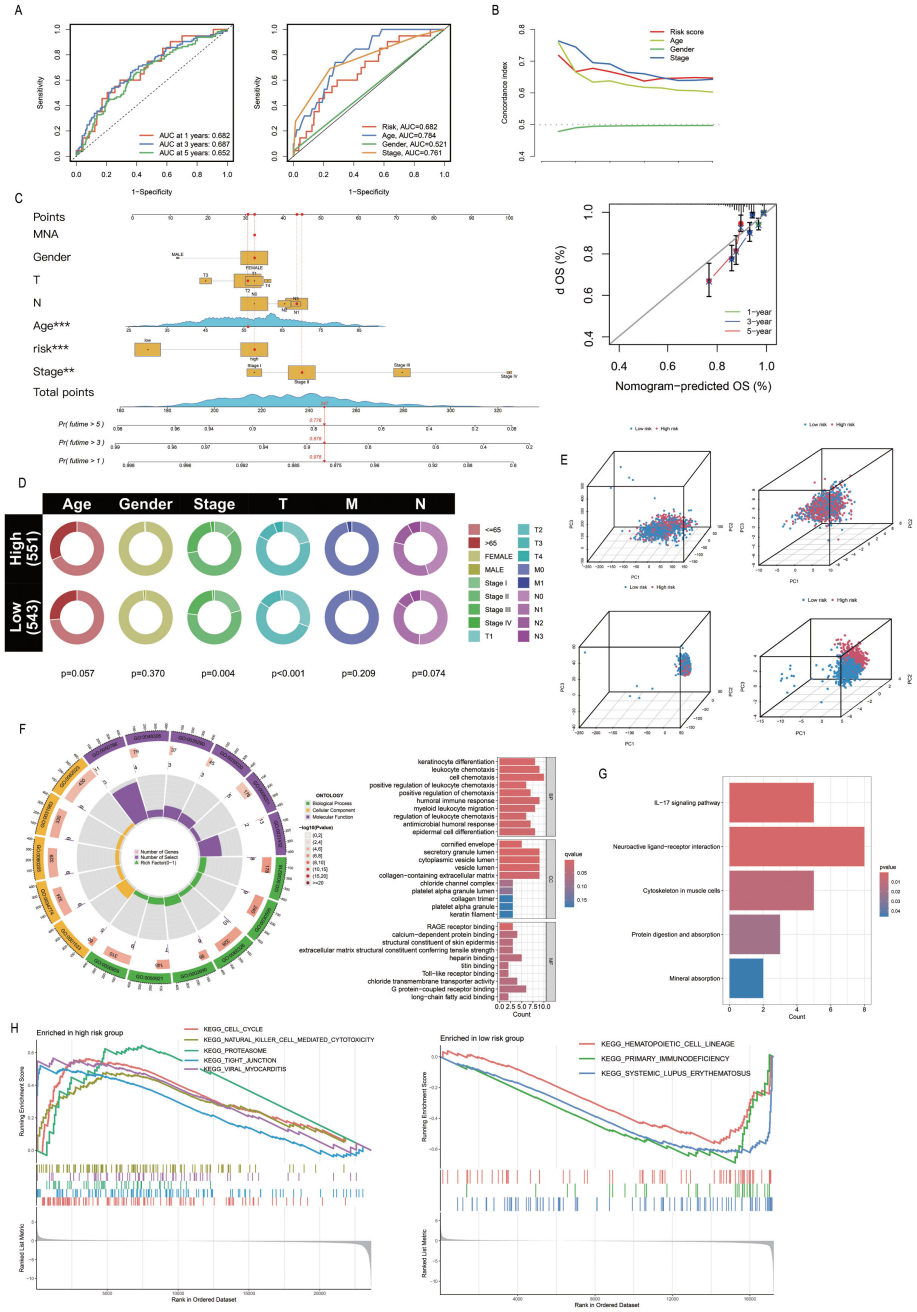
Association of PmPRLs with clinical characteristics and functional analysis. **(A)** The AUC values of the model were used to predict 1-year, 3-year and 5-year survival rates. **(B)** C-index analysis indicated that the predictive performance of the model in estimating patient survival rates was comparable to or even better than that of other clinical characteristics. **(C)** The nomogram and calibration curve were used to quantitatively score the prognosis of breast cancer (BC). **(D)** Correlation analysis of clinical characteristics revealed significant differences. **(E)** The nine PRLs used in model development showed significant discriminatory ability. **(F–H)** The results of GO, KEGG and GSEA enrichment analysis between different groups.

The PRL-based prediction model created in this study can be used to estimate the 1-year, 3-year, and 5-year survival rates of patients. The AUC value of the model exceeds 0.65 (AUC > 0.5), suggesting its robust predictive performance. Furthermore, by integrating the model with clinical features and validating it through ROC curve analysis, the results demonstrated that the model’s AUC was significantly higher than 0.5 (**Figure 4A**), confirming its high accuracy in predicting survival. Additionally, C-index analysis revealed that the model’s performance in survival prediction is comparable to or even surpasses traditional clinical features (**Figure 4B**). This study also constructed a nomogram to assist in survival prediction. By quantifying the scores of various clinical features and calculating the composite score, the survival probabilities of patients can be intuitively estimated. For instance, if the composite score of a sample is 247, the predicted survival probabilities for 1 year, 3 years, and 5 years are 0.978, 0.878, and 0.776, respectively. Calibration curve analysis further validated the high accuracy of the nomogram in predicting 1-year, 3-year, and 5-year survival periods (**Figure 4C**). Clinical correlation analysis showed significant differences between the “High-risk” group and the “low-risk” group in overall staging, T staging, and N staging (*P* < 0.05; **Figure 4D**), indicating that the risk stratification of the model has important clinical implications. Further validation of the model using the clinical dataset demonstrated its applicability to samples from different clinical groups (**Supplementary Figure S3**). The nine PRLs used for model development varied more significantly between the “High-risk” and “Low-risk” groups than the other PRLs, PALMs, and all lncRNAs. The PRLs used for model construction exhibited significant discriminative potential, distinguishing between the “High-risk” and “Low-risk” groups (**Figure 4E**). Analysis of risk differences revealed that 173 genes were differentially expressed between the “High-risk” and “Low-risk” groups (**Supplementary Table S5**). These DEGs were subjected to GO analysis, and the results were depicted using circle plots. The findings revealed that the DEGs were significantly enriched in various GO terms across the biological process (BP), cellular component (CC), and molecular function (MF) categories. These terms included the cell chemotaxis term in the BP category; secretory granule lumen, cytoplasmic vesicle lumen, vesicle lumen, and collagen-containing extracellular matrix terms in the CC category; and the G protein-coupled receptor binding term in MF (**Figure 4F**). KEGG enrichment analysis revealed that the neuroactive ligand-receptor interaction pathway was most significantly enriched and associated with the highest number of DEGs (**Figure 4G**). The results of GSEA revealed that the cell cycle, Natural Killer cell (NK)cell-mediated cytotoxicity, proteasome, tight junction, and viral myocarditis pathways were activated in the “High-risk” group, while hematopoietic cell lineage, primary immunodeficiency, and systemic lupus erythematosus were activated in the “Low-risk” group (**Figure 4H**).

### 3.5 Relationship between PmPRLs and immune function

**Figure 5:**
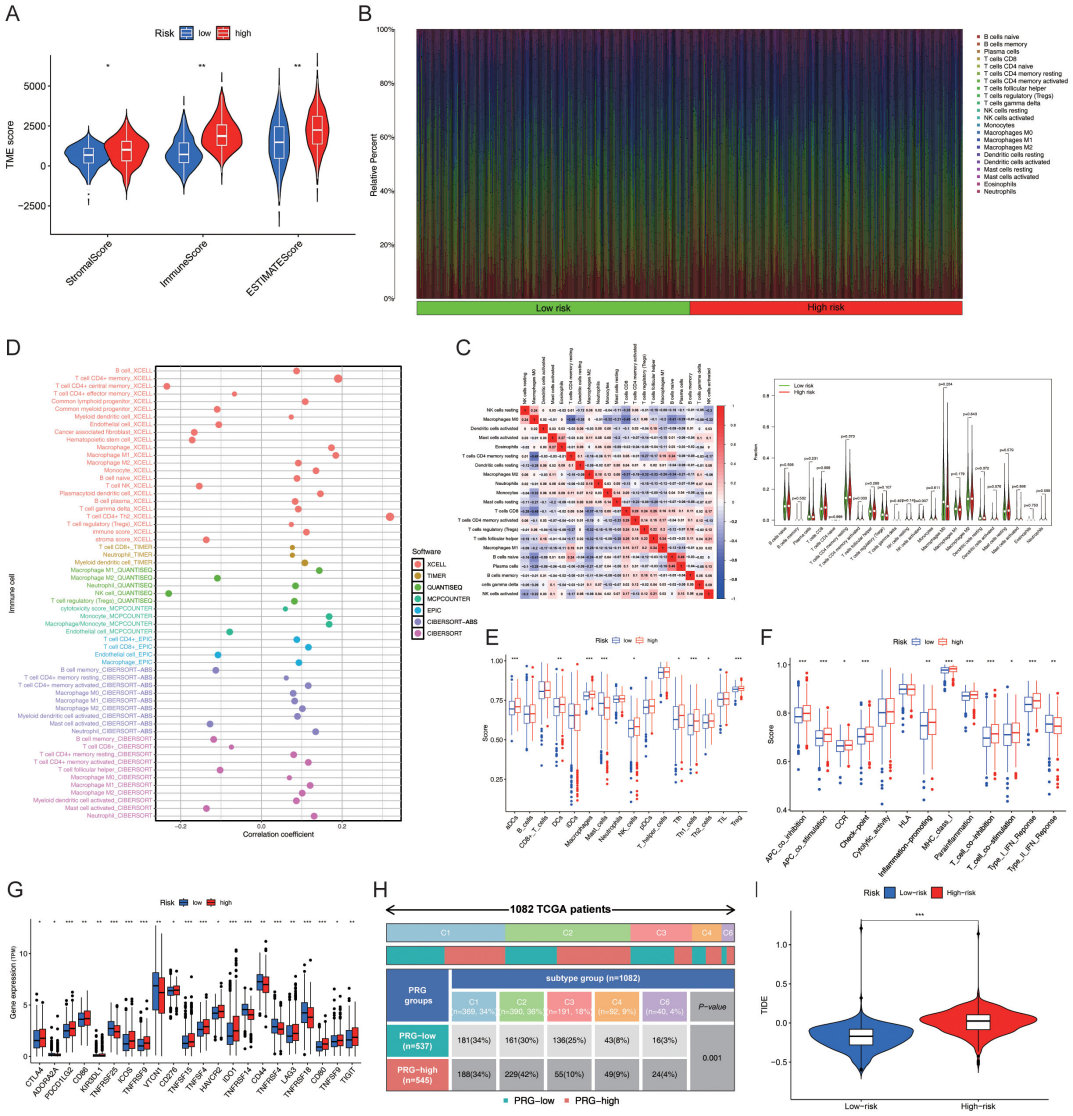
Prediction of immune functions using PmPRLs between the “high-risk” and “low-risk” groups **(A)** Comparison of the StromalScore, ImmuneScore, and ESTIMATEScore. **(B)** Composition of immune cells. **(C)** Differences in the abundance of CD4^+^ memory T cells and NK cells. **(D)** Further analysis using additional software identified CD4^+^ Th2 cells and CD4^+^ memory T cells as the key immune cell types that exhibited significant differences in abundance. **(E)** Significant differences in the immune functions of Th cells in the “High-risk” group, as revealed by ssGSEA. **(F)** T cell co-inhibition, APC co-stimulation, APC co-inhibition, checkpoint, para-inflammation, MHC class I, and type I IFN response were all significantly elevated in the “High-risk” group (*P* < 0.05). **(G)** Significant variations in key immune checkpoint-related genes (*P* < 0.05) and **(H)** immune typing of clinical traits (*P* < 0.05). **(I)** TIDE score in “High-risk” and “Low-risk” groups. *P < 0.05, **P < 0. 01, ***P < 0. 001.

The ESTIMATE package in R was used to calculate the StromalScore, ImmuneScore and ESTIMATEScore of the samples, and a differential analysis of the tumour microenvironment was conducted. The results showed that StromalScore, ImmuneScore and ESTIMATEScore were significantly different between the “High-risk” group and the “Low-risk” group (**Figure 5A**). The Cibersort package in R was used to analyse immune cell infiltration, and the abundance of each immune cell type in the samples was statistically analysed. The bar chart showed the differences in immune cell composition between the “High-risk” group and the “low-risk” group, as well as the positive and negative regulatory relationships among immune cells. The violin plot further depicted the significant differences in the abundance of CD4^+^ memory T cells and NK cells between the two groups (**Figures 5B-C**). Further analysis revealed that CD4^+^ Th2 cells and CD4^+^ memory T cells were the key differential immune cell types between the “High-risk” group and the “low-risk” group (correlation coefficients: 0.319 vs. 0.190; *P*-values:3.18E-27 vs. 2.30E-10) (**Figure 5D**). Subsequently, the GSVA package in R was used to evaluate the significant differences in immune function between the “High-risk” group and the “low-risk” group. Further ssGSEA revealed variations in the abundance of Th cells in the “High-risk” group (**Figure 5E**). T cell co-inhibition, APC co-stimulation, APC co-inhibition, checkpoints, para-inflammation, MHC class I, and type I IFN response were all significantly elevated in the “High-risk” group (*P* < 0.05; **Figure 5F**). The genes associated with key immune checkpoints, including *PDCD1LG2*, *TNFRSF25*, *ICOS*, *TNFRSF9*, *TNFRSF15*, *TNFRSF4*, and *CD80*, were differentially expressed between the “High-risk” and “Low-risk” groups (**Figure 5G**). The number and percentage of C1–C5 immune subtypes were compared between the “High-risk” and “Low-risk” groups for immune typing analysis. The chi-square test yielded a *P* value< 0.05, indicating significant differences in immune typing between the groups (**Figure 5H**). The efficacy of immunotherapy in the “High-risk” group and “Low-risk” group was evaluated based on the TIDE score retrieved from http://tide.dfci.harvard.edu/. Analysis of the violin plot revealed that the TIDE score was higher in the “High-risk” group, indicating a greater potential for immune escape and a poorer response to immunotherapy, which may contribute to poor prognosis (**Figure 5I**).

### 3.6 Association between model score and gene mutations

**Figure 6:**
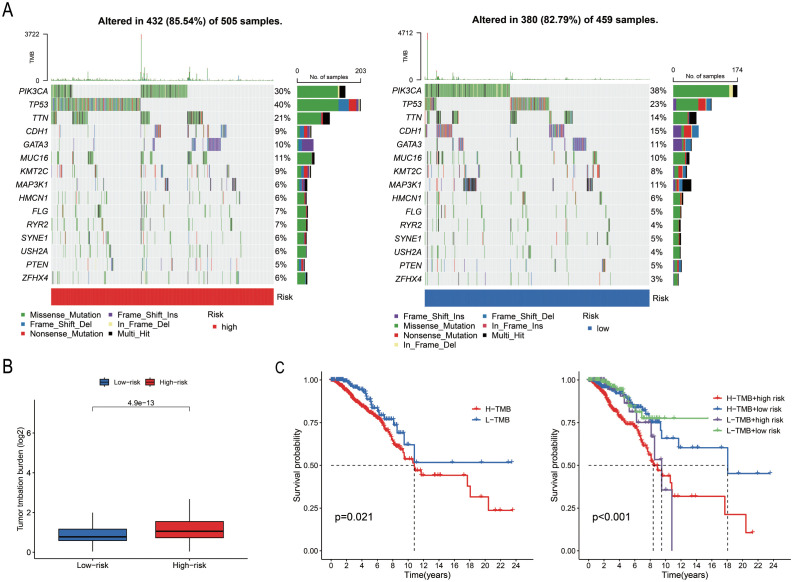
Analysis of somatic cell mutations based on model scores. **(A)** The waterfall plot illustrates the mutated genes and their mutation frequencies in the “High-risk” and “Low-risk” groups. **(B)** TMB levels in the two groups (*P* = 0.021). **(C)** Survival curves of the high TMB and low TMB groups, and the combined survival curve incorporating TMB levels and risk score (*P* = 0.001).

A total of 967 samples harbouring mutations were retrieved from TCGA-BRCA and categorized into the “High-risk” group and “Low-risk” groups based on the median risk score. The mutation frequency and tumour mutational burden (TMB) of the two groups were calculated using the maftools package in R. The mutation frequencies of *PIK3CA*, *CDH1*, *GATA3*, and *MAP3K1* were higher in the “Low-risk” group compared to those of the “Low-risk” group (**Figure 6A**). The TMB data and base mutation data of BC samples were retrieved from TCGA. A KM plot was generated to compare the survival probabilities of the high and low TMB groups, which revealed that the high TMB group exhibited a lower survival probability (*P* = 0.021; **Figure 6B**). The samples from TCGA were subsequently divided into four groups based on the level of risk (high or low) and TMB status, and the survival curves were fitted. The findings revealed that the “High-risk” and high TMB group exhibited the highest risk of BC (*P* < 0.001; **Figure 6C**).

### 3.7 Screening anti-cancer drugs against risk groups identified with PmPRLs

**Figure 7:**
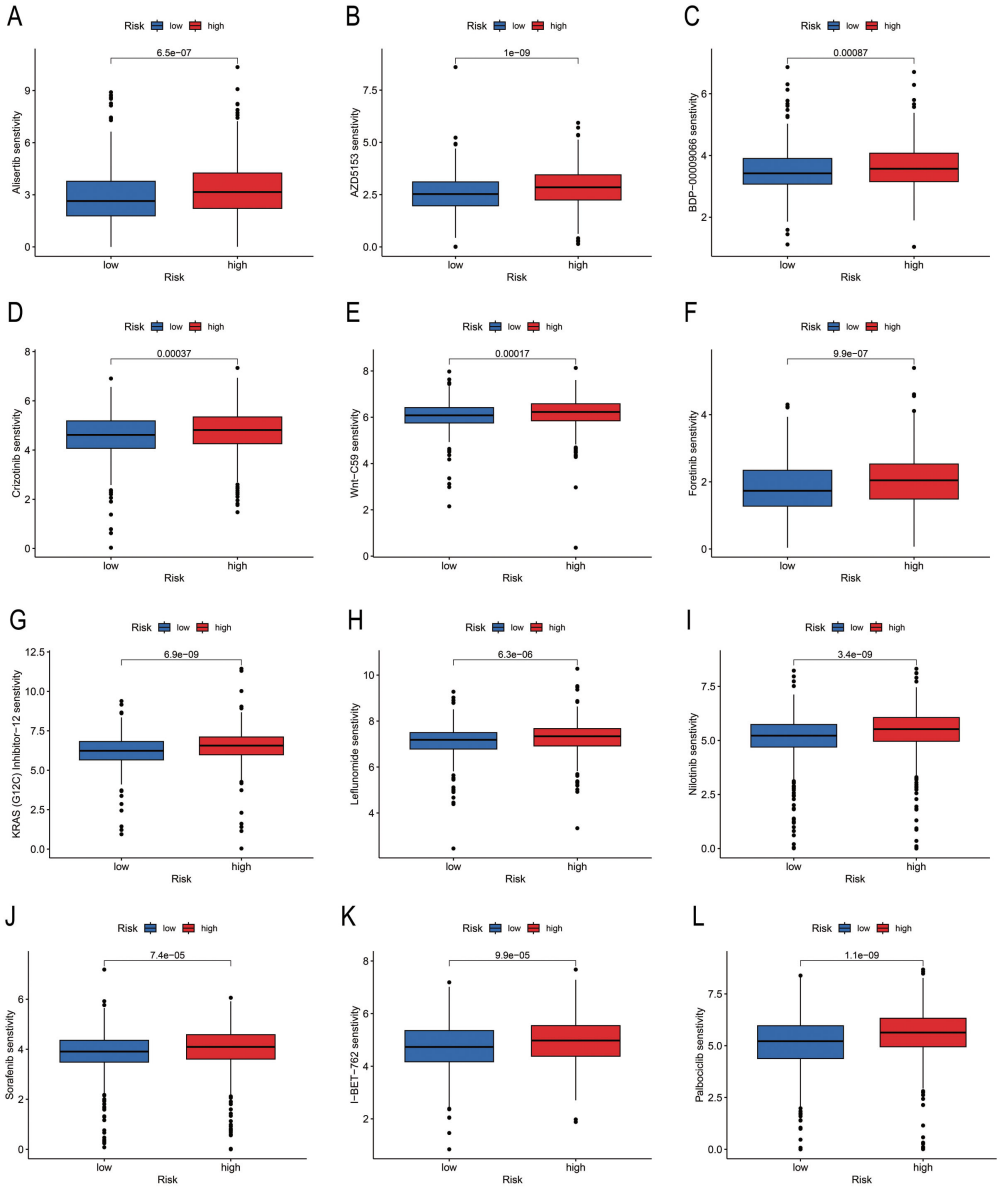
Differences in drug sensitivity of risk groups identified using PmPRLs. **(A–L)** Variations in the sensitivity of the different risk groups identified using PmPRLs to the 12 drugs for BC.

A total of 79 anti-cancer drugs with known sensitivity data (IC_50_) and significant associations with the developed model were screened using the “oncoPredict package” in R. Among the commonly used chemotherapy and targeted drugs for BC, we selected and analysed the sensitivity to 12 drugs. The findings demonstrated that the “Low-risk” group exhibited higher sensitivity to these drugs compared to the “High-risk” group (**Figure 7**).

### 3.8 Differential expression of PRLs for model development: correlation analysis

**Figure 8:**
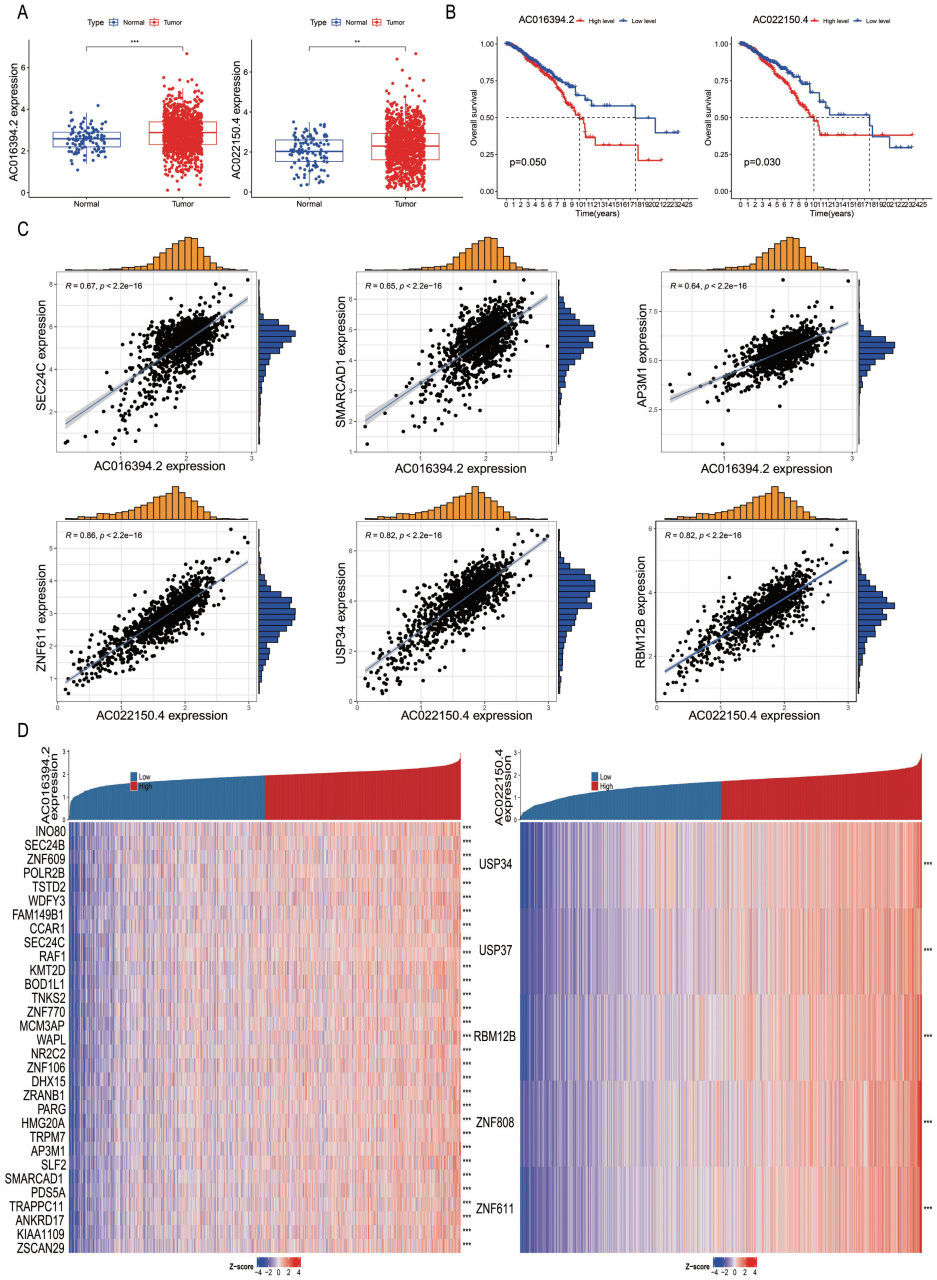
Correlation analysis of differentially expressed PRLs used for model development. **(A, B)** Analyses of the differential expression of AC016394.2 and AC022150.4 in tumour and adjacent tissues, and associated survival trends. **(C)** Correlation between the genes regulated by AC016394.2 (*SEC24C*, *SMARCAD1*, and *AP3M1*) and AC016394.2, as well as the correlation between the genes regulated by AC022150.4 (*ZNF611* and *USP34*) and AC022150.4. **(D)** Differences in the positive and negative regulatory relationships between the risk of BC associated with lncRNAs and various genes. **P < 0. 01, ***P < 0. 001.

Subsequently, we analysed the differential expression of nine PRLs used for model development in tumour tissues compared to adjacent normal tissues. The results showed that AC016394.2 and AC022150.4 were significantly upregulated in tumour tissues compared to adjacent normal tissues (**Figure 8A, P** < 0.05), while the expression levels of AC096642.2, AP000331.1, AC090510.2, AC011815.1, Z68871.1, AL109936.9, and AL162386.2 were significantly downregulated (**Supplementary Figure S4A**). Further analysis indicated that the low expression of AC016394.2 and AC022150.4 was associated with a higher overall survival rate (OS) (**Figure 8B**), and a similar trend was observed for AC096642.2 (*P* = 0.044) and Z68871.1 (*P* = 0.025). In contrast, the high expression of AP000331.1 (*P* = 0.036), AC011815.1 (*P* = 0.026), and AL109936.9 (*P* = 0.005) was associated with a higher OS. However, the expression levels of AC090510.2 and AL162386.2 were not significantly correlated with OS (*P* > 0.05; **Supplementary Figure S4B**). Interestingly, the external dataset TANRIC confirmed the high expression of AC022150.4 in tumours compared to normal cells (*P*<0.05; **Supplementary Figure S4C**). Based on these findings, we selected AC016394.2 and AC022150.4 for further study. Through co-expression analysis, we initially identified the genes regulated by these two lncRNAs and screened out differentially expressed genes (DEGs) with correlations (R) greater than 0.6 and 0.8. Ranked in the descending order of correlation, the genes regulated by AC016394.2 were *SEC24C*, *SMARCAD1*, and *AP3M1*, while the genes regulated by AC022150.4 included *ZNF611* and *USP34* (**Figure 8C**). The expression profiles of the other related genes with weak correlation are depicted in **Supplementary Figure S5**. The co-expression patterns of AC016394.2 and AC022150.4 were visualized using a heat map, which revealed that an increase in the expression of these lncRNAs was associated with both positive and negative regulatory relationships between different genes and target lncRNAs (*P* < 0.01; **Figure 8D**).

### 3.9 Prediction of immune functions of *SEC24C* and *ZNF611* at single-cell level

**Figure 9:**
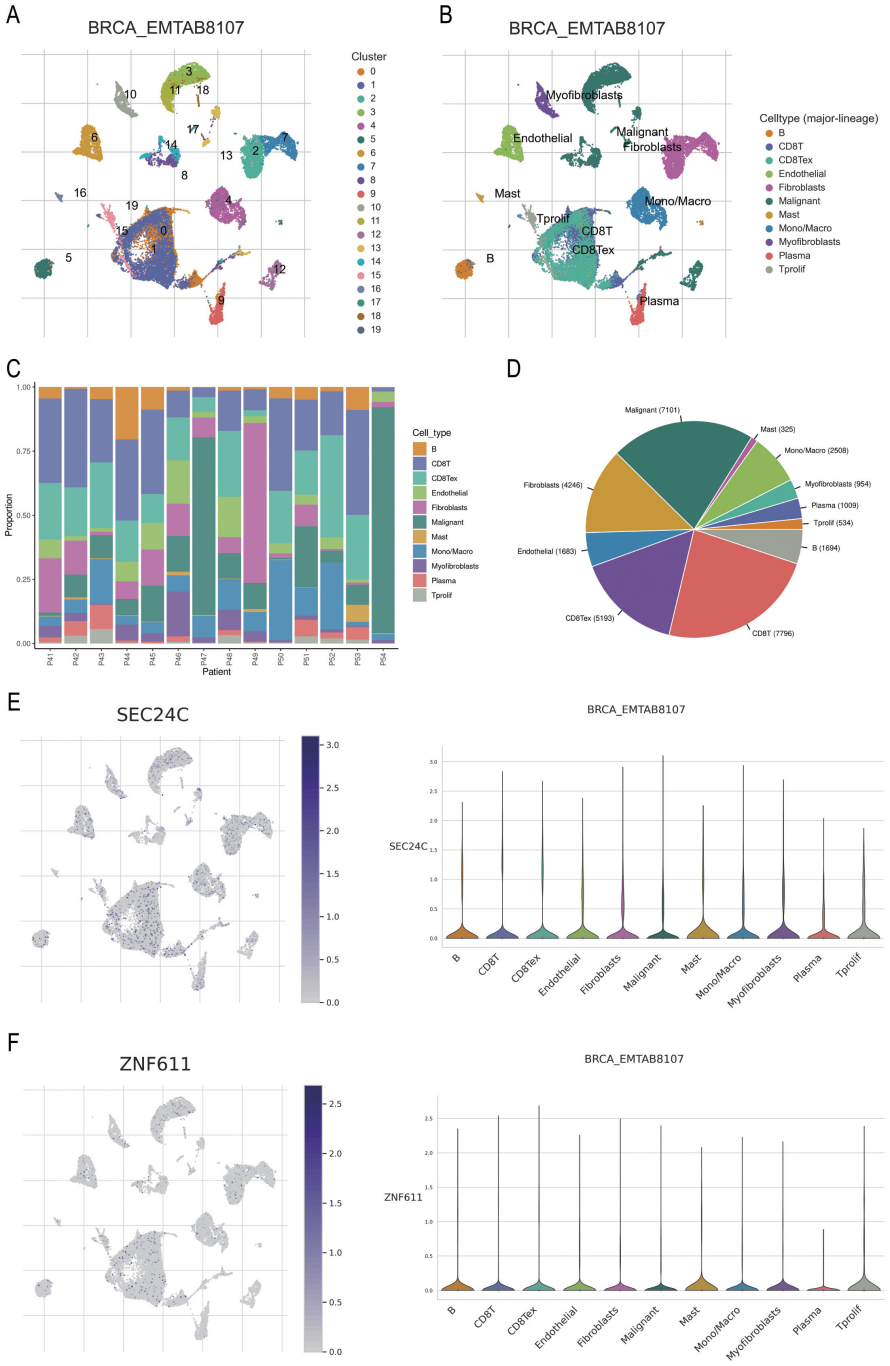
Prediction of the potential immune roles of *SEC24C* and *ZNF611* at the single-cell level. **(A, B)** Clustering and annotation of the BRCA_EMTAAB8107 BC dataset. **(C, D)** Malignant and CD8+ T cells were the most abundant cell types in the dataset. **(E, F)** *SEC24C* and *ZNF611* were predominantly localized in mast cells.

The BRCA_EMTAAB8107 BC gene set was retrieved from http://tisch.compbio.cn/ and subsequently divided into 19 clusters (**Figure 9A**). The distribution of various immune cell types was analysed following cell annotation (**Figure 9B**). The findings revealed that the proportion of malignant and CD8^+^ T cells was the highest (**Figures 9C–D**), while *SEC24C* and *ZNF611* were predominantly localized in mast cells (**Figures 9E–F**).

### 3.10 Effects of AC016394.2 and AC022150.4 on cell growth, proliferation, and migration

**Figure 10:**
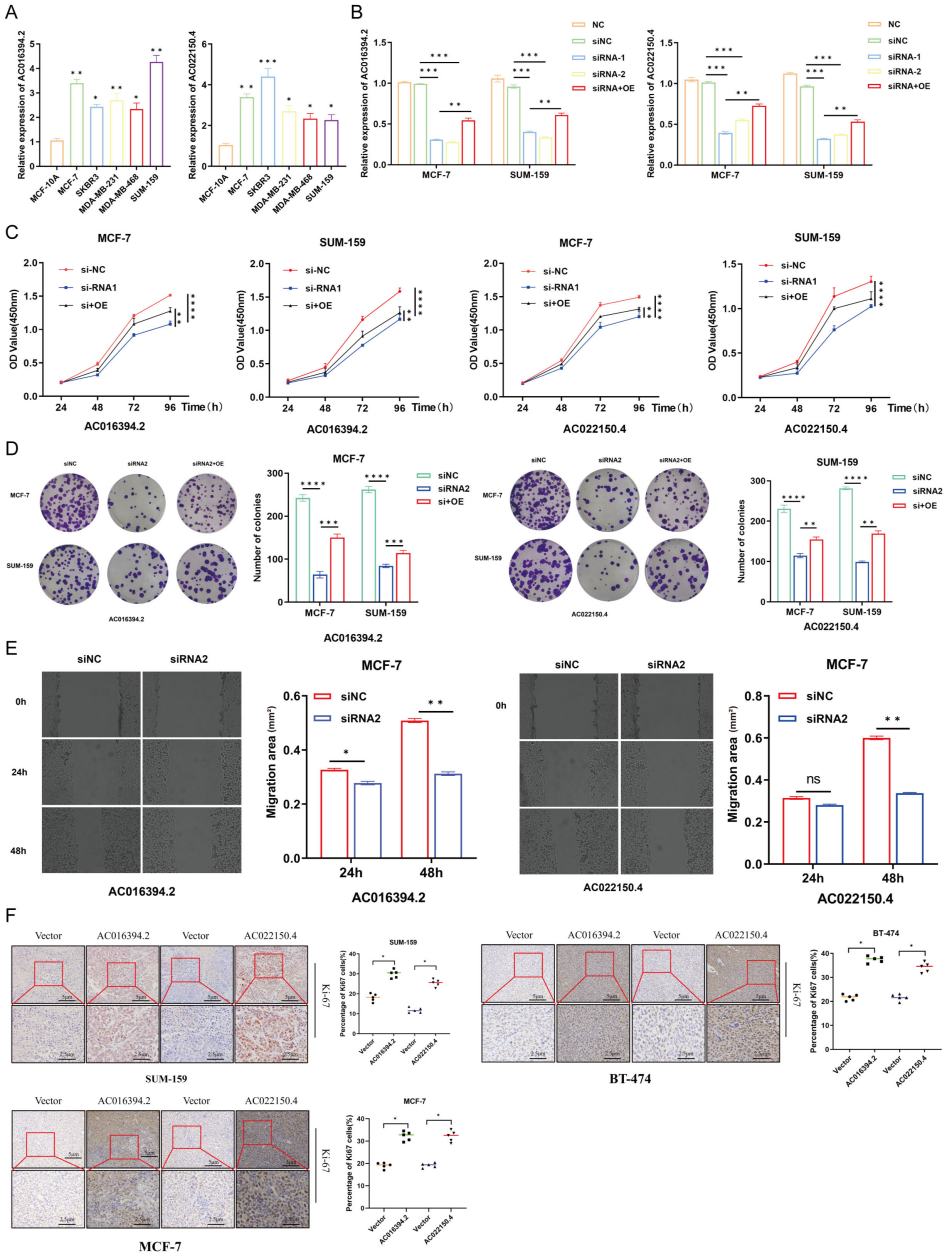
Functional verification of LncRNA AC016394.2 and AC022150.4. **(A)** Expression of lncRNAs in normal breast epithelial cells and different subtypes of BC cells. **(B)** Expression of AC016394.2 and AC022150.4 after the process of siRNA and siRNA+OE. **(C)** Proliferation rates of MCF-7 and SUM-159 cells at different time points following treatment with siRNAs and siRNA+OE targeting AC016394.2 and AC022150.4. **(D)** Alterations in cell growth following treatment with siRNAs and siRNA+OE targeting AC016394.2 and AC022150.4. **(E)** The migration rate of MCF-7 cells treated with siRNAs targeting AC016394.2 and AC022150.4 was lower than that of the control group after 48 (h) **(F)** Assessment of Ki-67 expression in cells overexpressing AC016394.2 and AC022150.4 by IHC analysis. *P < 0.05, **P < 0.01, ***P < 0.005, ****P < 0.001, ns, not significant (*P* > 0.05).

The total RNA was extracted from the six breast cell lines, including normal MCF-10A breast epithelial cells, luminal-type MCF-7 cells, Her-2-type SKBR3 cells, and TNBC-type MDA-MB-231, MDA-MB-468, and SUM-159 cells, and subjected to qPCR analysis. The results demonstrated that the two PRLs used for model development, AC016394.2 and AC022150.4, were markedly differentially expressed across the different subtypes of BC cell lines, which was consistent with the results of earlier bioinformatics predictions (**Figure 10A**). The MCF-7 and SUM-159 cells were subsequently transfected with overexpression and siRNA plasmids constructed for the overexpression and knockdown of AC016394.2 and AC022150.4, and the findings revealed that the expression of AC016394.2 and AC022150.4 was significantly affected (**Figure 10B**). CCK-8 proliferation assays revealed that the proliferation of MCF-7 and SUM-159 cells was significantly activated/inhibited at the 96-h mark following treatment with the overexpression/siRNAs (**Figure 10C**). Clone formation assays additionally demonstrated that treatment with siRNAs targeting AC016394.2 and AC022150.4 inhibited cell growth but acquired rescue after overexpression (**Figure 10D**). The treatment of MCF-7 cells with siRNAs targeting AC016394.2 and AC022150.4 significantly reduced the rate of migration after 48 h, compared to that of the control group (**Figure 10E**). Following surgery, the SUM-159 (TNBC), BT-474 (HER2+), and MCF-7 (Luminal) cells overexpressing AC016394.2 and AC022150.4, along with control cells harbouring the vector, were xenografted into BALB/c nude mice. The mice were euthanised after 14 days and the tumours were removed. Histopathological sections were prepared and stained, and subsequent IHC analysis revealed that the nuclear expression of Ki-67 was significantly upregulated (**Figure 10F**).

### 3.11 The relationship between these two lncRNAs and palmitoylation

**Figure 11:**
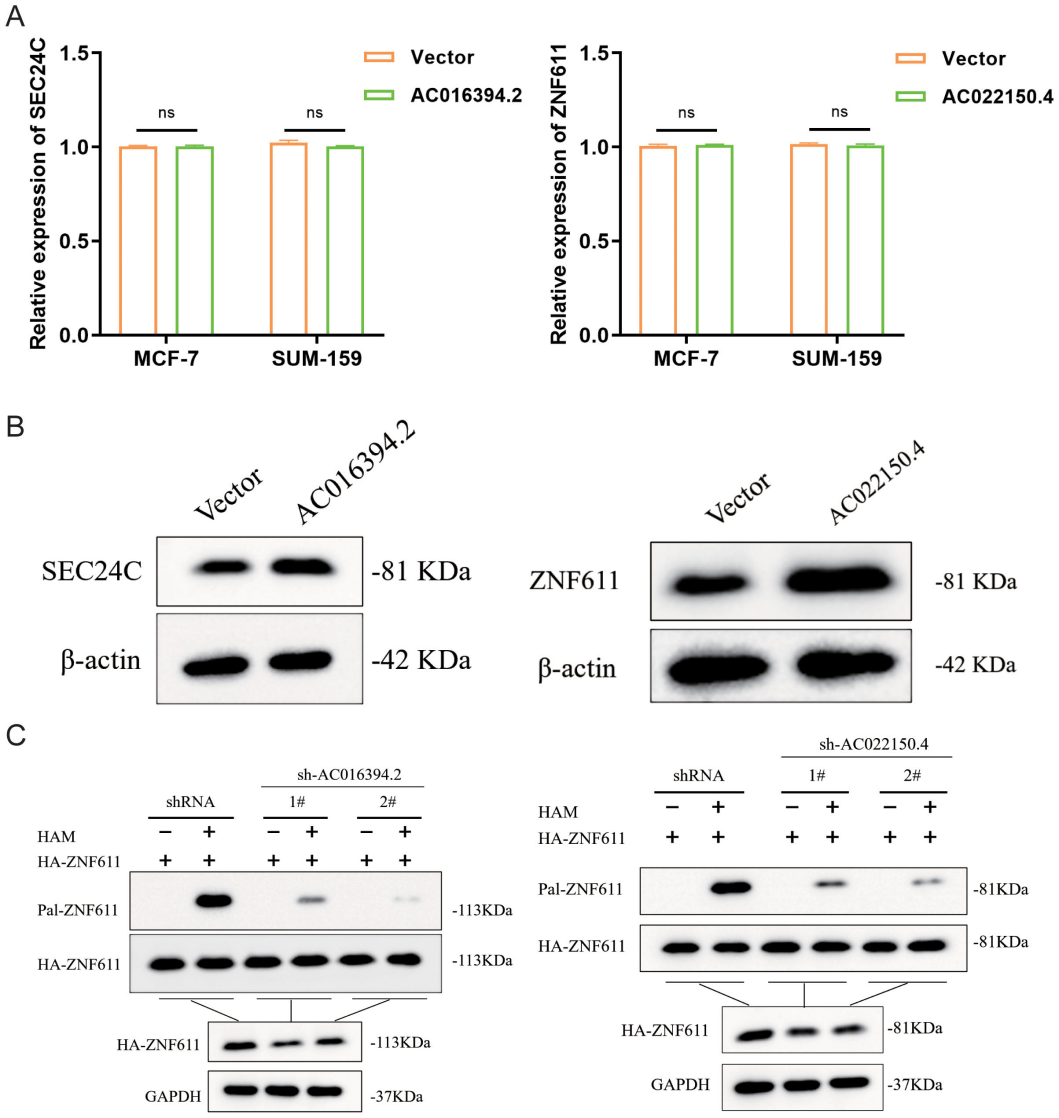
The role of LNCRNA in palmitoylation modification of co-expressed proteins. **(A)** The expression levels of both LncRNA and target gene Mrna. **(B)** The protein expression status of their interacting proteins after overexpression of two LncRNAs respectively. **(C)** SEC24C and ZNF611 palmitoylation in sh-LncRNA lentivirus–infected HEK293T cells analysed by ABE assay. ns, not significant (*P*> 0.05).

Based on the previous co-expression analysis, it has been established that the gene regulated by AC016394.2 is primarily SEC24C, while the gene regulated by AC022150.4 is predominantly ZNF611. Following the lysis of SUM-159 cells, the supernatant was collected for further analysis. The expression levels of LncRNA and target gene mRNA were assessed using quantitative PCR (qPCR), revealing no significant differences in expression levels (**Figure 11A**). Subsequently, overexpression vectors for AC016394.2 and AC022150.4 were transfected into target cells, leading to a marked increase in protein expression levels of SEC24C and ZNF611, as determined by Western Blot analysis(**Figure 11B**). This suggests that LncRNA may have facilitated post-translational modifications of these target proteins. To investigate palmitoylated proteins, ABE (Acyl-Biotin Exchange) assays were conducted with interference from AC016394.2/AC022150.4 knockdown experiments. The results indicated that silencing AC016394.2 significantly diminished the palmitoylation of SEC24C, whereas knockdown of AC022150.4 resulted in decreased palmitoylation of ZNF611 (**Figure 11C**).

## 4 Discussion

PRL can regulate various stages of transcription, for instance, by forming a triple helix structure with DNA and interfering with the binding of transcription factors to DNA. Unlike mRNAs, lncRNAs can directly regulate gene expression at the transcription initiation stage. Some PRLs can act as tumour suppressors or promoters, thereby controlling the proliferation, invasion, and metastasis of tumour cells. These findings suggest that therapeutic intervention targeting lncRNAs involved in protein palmitoylation may have higher specificity and fewer side effects, providing new strategies and directions for the treatment of various malignant tumours. Therefore, this study identified 592 PRLs and constructed a predictive model (PmPRL) based on their risk levels to assess the prognosis of breast cancer (BC). The training set was validated using an internal validation set based on clinical features, and the results demonstrated that PmPRL could serve as an independent prognostic model. Subsequently, patients were divided into “High-risk” and “low-risk” groups based on PmPRL, and differentially expressed genes (DEGs) between the two groups were analysed, ultimately identifying 173 DEGs. Further gene set enrichment analysis (GSEA) revealed that these genes exhibited significantly different activities between the “High-risk” and “low-risk” groups. Patients in the “High-risk” group had lower survival rates and decreased drug sensitivity. Additionally, immune cell and immune microenvironment analysis indicated significant differences in Th cell immune function in the “High-risk” group. Moreover, multiple immune-related pathways were activated, including T cell co-inhibition, antigen-presenting cell (APC) co-stimulation, APC co-inhibition, checkpoints, anti-inflammatory responses, MHC class I molecule expression, and type I IFN responses. Through in-depth exploration of the nine PRLs involved in the model development process, two key lncRNAs, AC016394.2 and AC022150.4, were identified. Co-expression analysis revealed that SEC24C and ZNF611 were highly correlated with AC016394.2 and AC022150.4, respectively, and predicted their potential roles in immune function. The research results showed that these two genes were mainly located in mast cells. *In vitro* and *in vivo* experiments further confirmed that both AC016394.2 and AC022150.4 could promote the proliferation and migration of breast cancer cells, laying the foundation for further investigation of the molecular mechanisms of these two lncRNAs.

It is worth noting that recent studies have shown that mast cells play a crucial role in the anti-tumour immunity of triple-negative breast cancer (TNBC). Moreover, there is evidence that the functional activation of mast cells in combination with PD-L1 inhibitors can significantly inhibit the growth and progression of TNBC tumours. Therefore, mast cells may become potential targets for enhancing the efficacy of immunotherapy (12). Given that SEC24C and ZNF611 are regulated by lncRNAs AC016394.2 and AC022150.4, respectively, it is speculated that they may regulate immune function by influencing the activity of mast cells.

Lu et al. reported that AC016394.2 can act as a copper death-related lncRNA, and its differential expression can be used to predict the functional characteristics of prostate cancer (13). The study by Xing et al. revealed that AC016394.2 can also function as a disulfide death-related lncRNA, and can be used to predict the prognosis of gastric cancer (14). Another study demonstrated that the AC022150.4 lncRNA likely holds predictive potential for assessing lipid metabolism in BC (15). AC022150.4 can also function as a blood exosome-related lncRNA and act as a prognostic predictor in small cell lung cancer (16).

The present study has one limitation, namely, all the data pertaining to BC were retrieved from TCGA, which may have introduced potential biases.

In summary, the present study established a novel PRL signature in BC and experimentally validated the predictive potential of the PRLs, AC016394.2 and AC022150.4, in estimating the prognosis of BC, and their verification of palmitoylation modification in co-expressed proteins.

## 5 Conclusion

The present study identified two novel PRLs that are likely associated with the prognosis of BC. By screening the “High-risk” and “Low-risk”groups for drug sensitivity, the study identified potential therapeutic targets for the treatment of BC in these groups. Given the critical roles of lncRNAs in cellular physiology, immune function, and other cellular processes, the findings offer novel insights for increasing the survival rate of patients with BC.

## Data Availability

The original contributions presented in the study are included in the article/**Supplementary Material**. Further inquiries can be directed to the corresponding author/s.

## Supplementary material

Supplementary Figure 1 Flowchart depicting the research methodology for PRL-based model development.

Supplementary Figure 2 Classification of tumor samples using PmPRLs. The tumor samples were classified based on the expression levels of PRLs used for model development, and were divided into three subtypes. The BC tumor samples were classified using PmPRLs. Among the tested values, κ = 3 exhibited a flatter distribution and was more closely aligned with the maximum Cumulative Distribution Function (CDF). **A** Based on this, κ = 3 was selected for classification, and the BC tumor samples were categorized into three subtypes, namely, cluster 1 (C1), cluster 2 (C2), and cluster 3 (C3). **B** Survival analysis indicated that the prognosis of C1 was poorer than that of C1 and C3. **C** The relationship between tumor subtypes and the “High-risk” and “Low-risk” groups was illustrated using the Sankey diagram, which revealed that C3 and C1 primarily consisted of patients with low and high risk scores, respectively. **D** Analysis of the tumor microenvironment of classified tumors. The stromal cell scores varied between C1 and C2. Differences in immune cell scores between C1 and C2, and between C2 and C3. Differences in the ESTIMATEScore between C2 and C3. The findings indicate the potential of PmPRLs in classifying BC tumors and risk stratification. **E** Analysis of immune cell types in the three subtypes, as determined by various software. **F** Analysis of immune checkpoints across the classified subtypes revealed differences among C1, C2, and C3. **:P < 0.05*, ***:P<0.01*, ****:P<0. 001*.

Supplementary Figure 3 Survival curves of different clinical subgroups based onrisk scores. The model was applicable to samples from different clinical groups, primarily showing survival differences in terms of **A** age (>65 (*P* = 0.007) and ≤65 (*P* < 0.001); **B** sex (female; *P* < 0.001), **C** overall stage (*P* < 0.001), **D** T stage (*P* < 0.001), and **E** N stage (*P* < 0.05). **F** The model was not applicable to male patients (*P* = 0.540) and those with M1 stage tumors (*P* = 0.860).

Supplementary Figure 4 Expression and survival plots of the seven lncRNAs used for modeling. **A** The lncRNAs AC096642.2, AP000331.1, AC090510.2, AC011815.1, Z68871.1, AL109936.9, and AL162386.2 were expressed at low levels. **B** Low expression levels of AC096642.2 (*P* = 0.044) and Z68871.1 (*P* = 0.025) were associated with higher OS. Conversely, high expression levels of AP000331.1 (*P* = 0.036), AC011815.1 (*P* = 0.026), and AL109936.9 (*P* = 0.005) were associated with higher OS, while high or low expression levels of AC090510.2 and AL162386.2 were not associated with significant differences in OS (*P* > 0.05). C. The external dataset TANRIC confirmed the differential expression of AC022150.4 between tumor and normal cells (*P*<0.05). **:P < 0.05*, ***:P<0. 01*, ****:P<0. 001*.

Supplementary Figure 5 Genes co-expressed with AC016394.2 and AC022150.4. **A** Correlation between AC016394.2 and the co-expressed genes, *INO80*, *TNKS2*, *WAPL*, and *ZSCAN29*. **B** Correlation between AC022150.4 and the co-expressed genes, *USP37* and *ZNF808*.

## Glossary

PPT1: Palmitoyl - protein thioesterase 1
APT1: Protein acyl – thioesterases 1
APT2: Protein acyl – thioesterases 2
ABHD17A: Abhydrolase Domain Containing 17A
ABHD17B: Abhydrolase Domain Containing 17B
ABHD17C: Abhydrolase Domain Containing 17C
FASN: Fatty Acid Synthase
YTHDF3: YTH Domain Family Protein 3
MYC: Myelocytomatosis Oncogene
CLOCK: Circadian Locomotor Output Cycles Kaput
CircPDIA3: Circular RNA - Protein Disulfide - Isomerase A3
GSDME: Gasdermin E
IFNGR1: Interferon - gamma Receptor 1
TIM – 3: T -cell immunoglobulin and mucin - domain molecule-3
CAR-T: Chimeric Antigen Receptor T – cell
DUXAP8: Double Homeobox A Pseudogene 8
SLC7A11: Solute Carrier Family 7 Member 11
PCA: Principal component analysis
MHC: Major Histocompatibility Complex
IFN: Interferon
PDCD1LG2: Programmed Cell Death 1 Ligand 2
TNFRSF25: Tumour Necrosis Factor Receptor Superfamily Member 25
ICOS: Inducible T - cell Co – stimulator
TNFRSF9: Tumour Necrosis Factor Receptor Superfamily Member 9
TNFRSF15: Tumour Necrosis Factor Receptor Superfamily Member 15
TNFRSF4: Tumour Necrosis Factor Receptor Superfamily Member 4
CD80: Cluster of Differentiation 80
TMB: Tumour mutational burden
PIK3CA: Phosphatidylinositol - 4,5 - bisphosphate 3 - kinase, catalytic subunit alpha
CDH1: Cadherin – 1
GATA3: GATA - binding protein 3
MAP3K1: Mitogen - activated protein kinase kinase kinase 1
SEC24C: SEC24 Homolog C, COPII Coat Complex Component
SMARCAD1: SNF2 Related Chromatin Remodelling ATPase With DExD Box 1
AP3M1: Adaptor Related Protein Complex 3 Subunit Mu 1
ZNF611: Zinc Finger Protein 611
USP34: Ubiquitin Specific Peptidase 34
TNBC: Triple-negative Breast Cancer
PD-L1: Programmed Death - Ligand 1
CDF: Cumulative Distribution Function
INO80: INO80 complex ATPase subunit
TNKS2: Tankyrase 2
WAPL: WAPL cohesin release factor
ZSCAN29: Zinc Finger and SCAN Domain Containing 29
USP37: Ubiquitin Specific Peptidase 37
ZNF808: Zinc Finger Protein 808
