# Supplementary figures and images for: Construction of a prognostic model based on palmitoylation-related lncRNAs for assessing drug benefits in breast cancer
Source: Front Immunol. 2025 Oct 27;16:1656593. doi: 10.3389/fimmu.2025.1656593 (PMC12597904; doi:10.3389/fimmu.2025.1656593)

Fig.S1

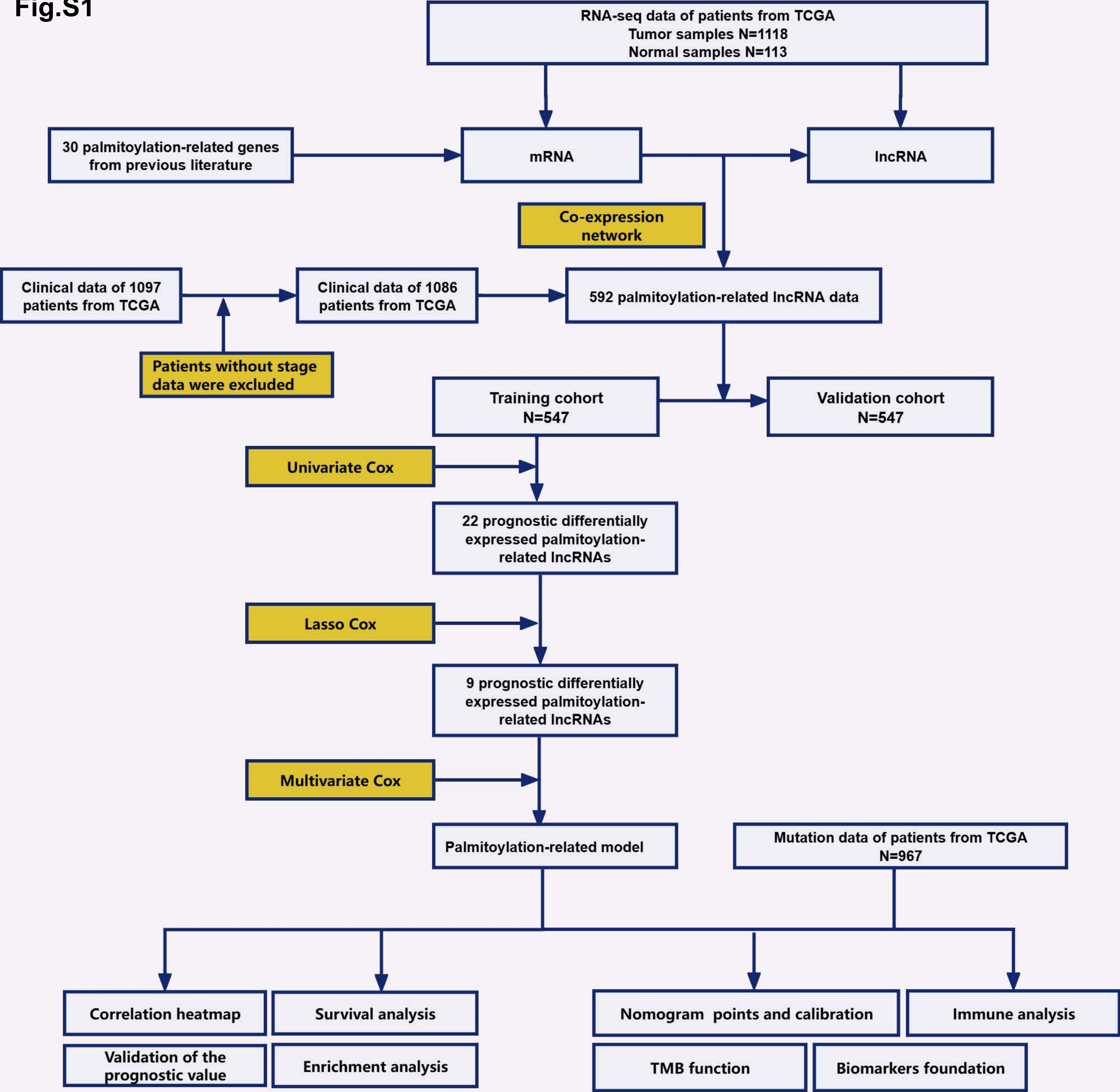

Supplement: Supplementary Figure 1 — Flowchart depicting the research methodology for PRL-based model development. [file Image1.pdf]

A

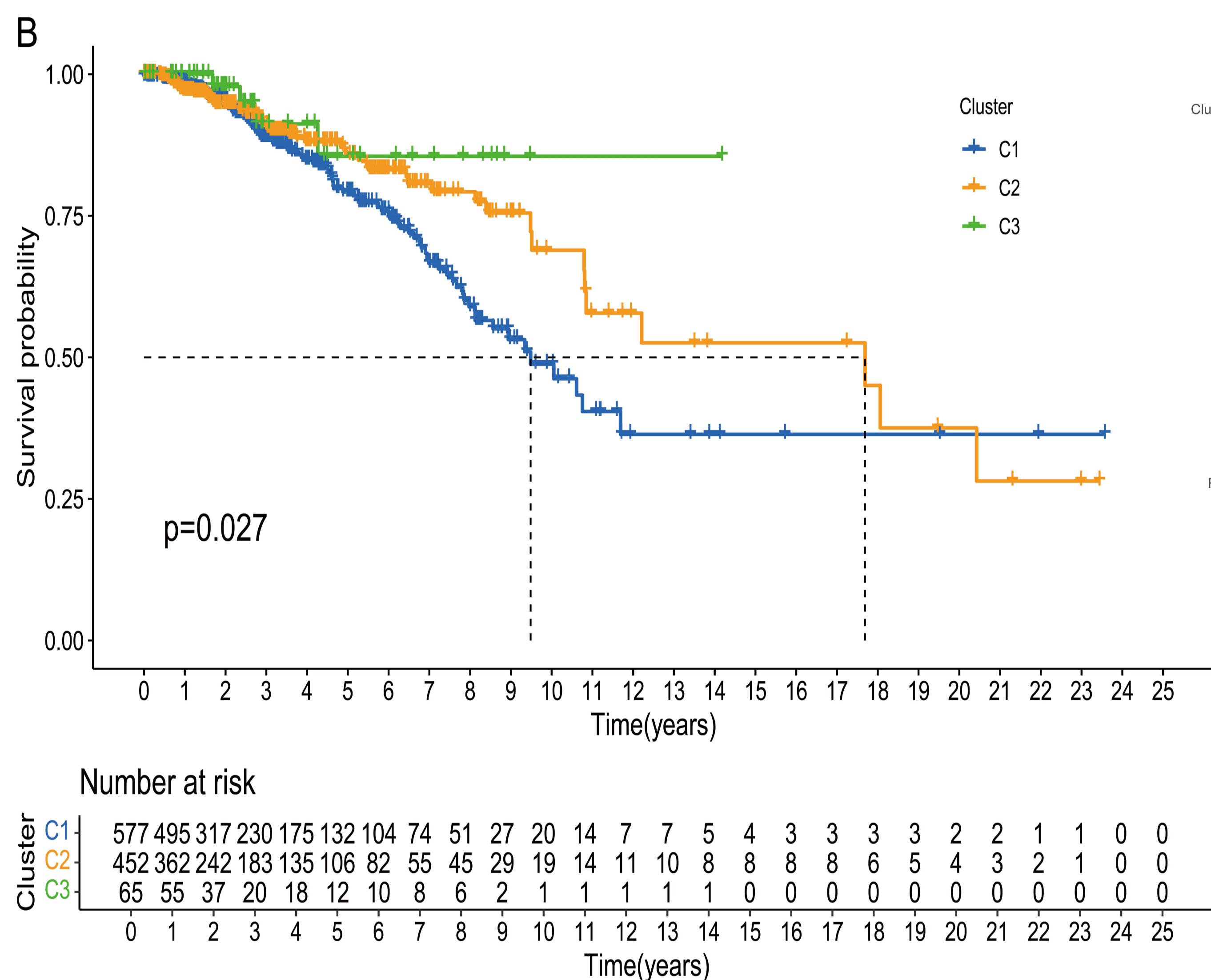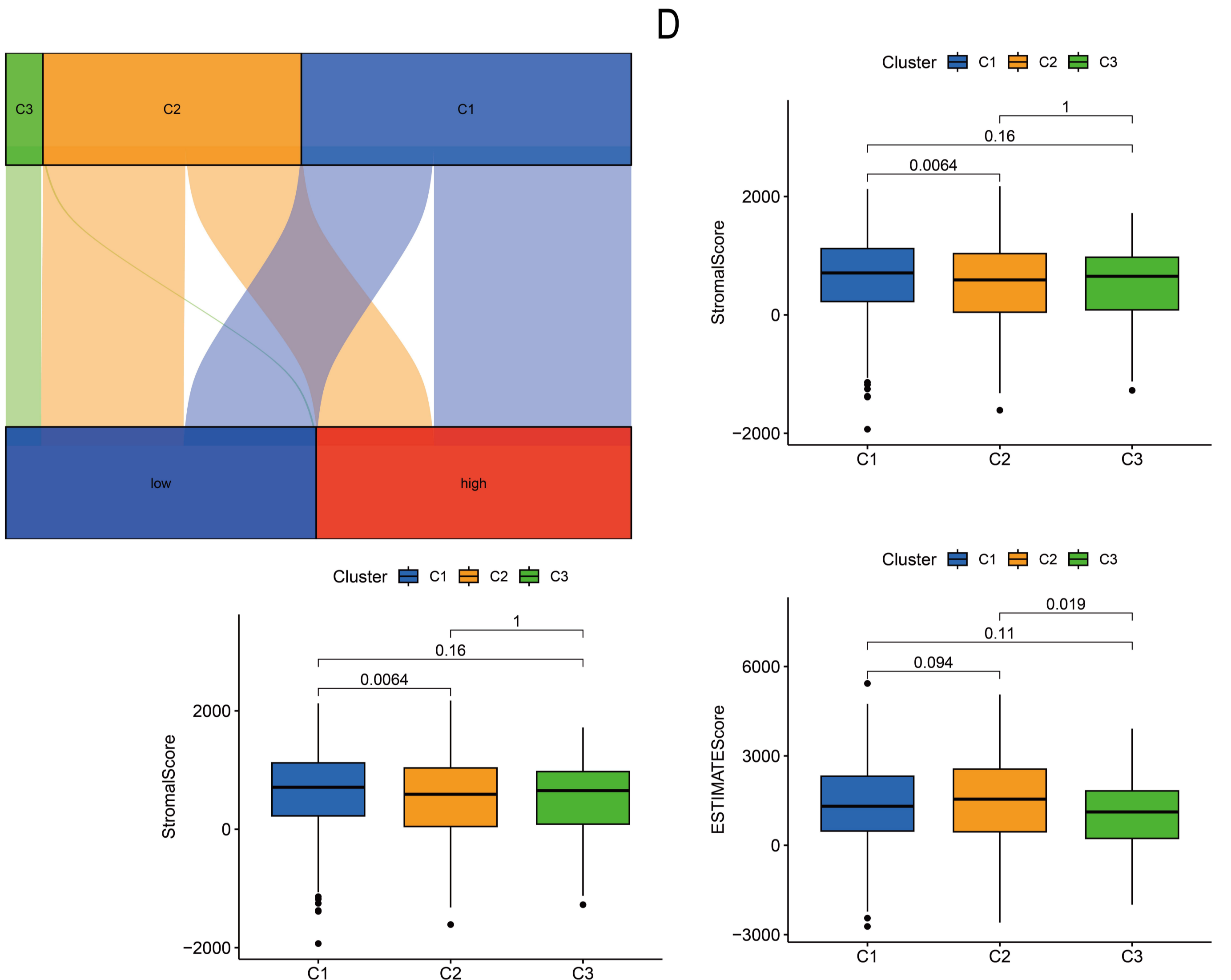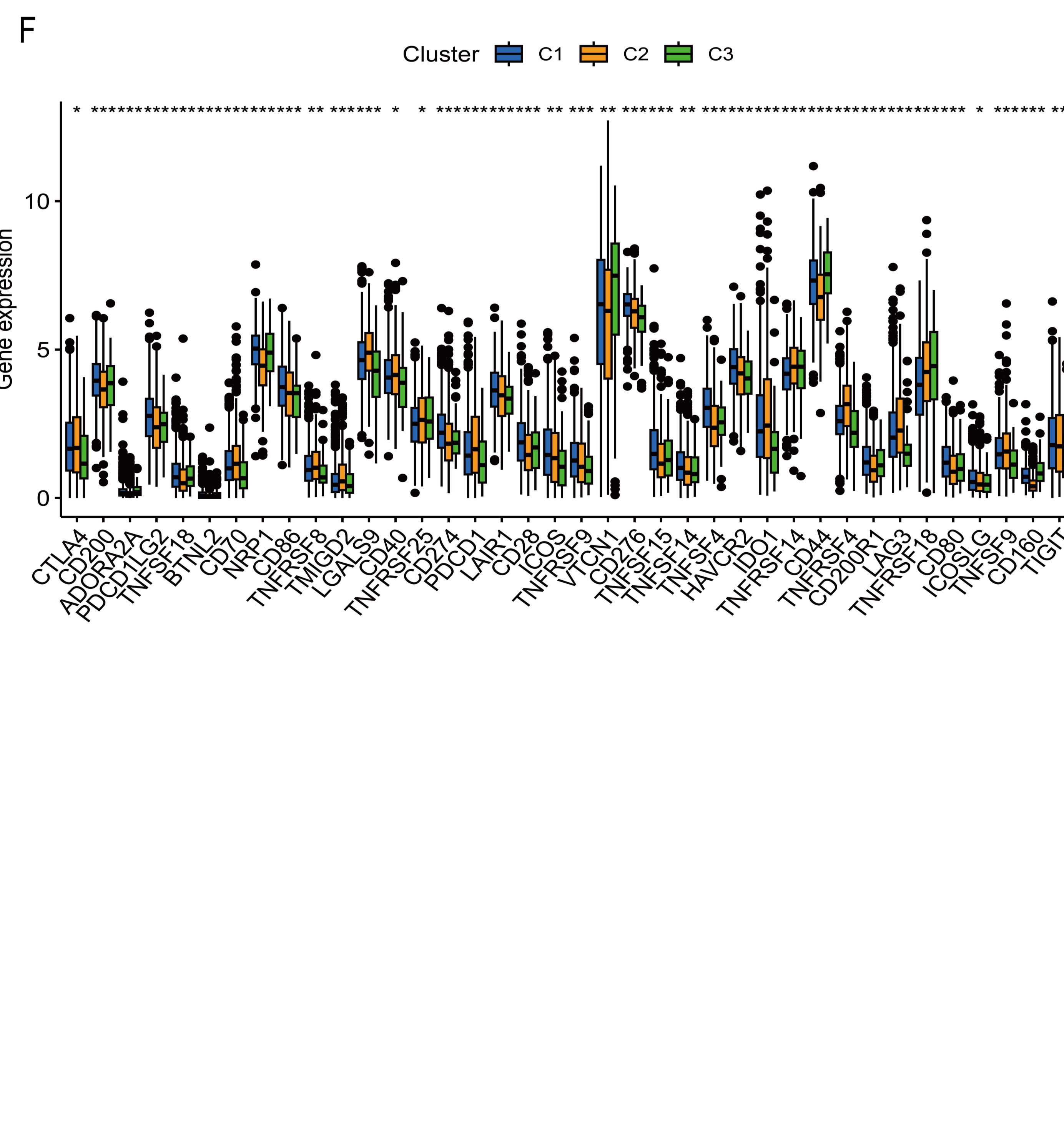

Supplement: Supplementary Figure 2 — Classification of tumor samples using PmPRLs. The tumor samples were classified based on the expression levels of PRLs used for model development, and were divided into three subtypes. The BC tumor samples were classified using PmPRLs. Among the tested values, κ = 3 exhibited a flatter distribution and was more closely aligned with the maximum Cumulative Distribution Function (CDF). A Based on this, κ = 3 was selected for classification, and the BC tumor samples were categorized into three subtypes, namely, cluster 1 (C1), cluster 2 (C2), and cluster 3 (C3). B Survival analysis indicated that the prognosis of C1 was poorer than that of C1 and C3. C The relationship between tumor subtypes and the “High-risk” and “Low-risk” groups was illustrated using the Sankey diagram, which revealed that C3 and C1 primarily consisted of patients with low and high risk scores, respectively. D Analysis of the tumor microenvironment of classified tumors. The stromal cell scores varied between C1 and C2. Differences in immune cell scores between C1 and C2, and between C2 and C3. Differences in the ESTIMATEScore between C2 and C3. The findings indicate the potential of PmPRLs in classifying BC tumors and risk stratification. E Analysis of immune cell types in the three subtypes, as determined by various software. F Analysis of immune checkpoints across the classified subtypes revealed differences among C1, C2, and C3. *:P < 0.05, **:P<0.01, ***:P<0. 001. [file Image2.pdf]

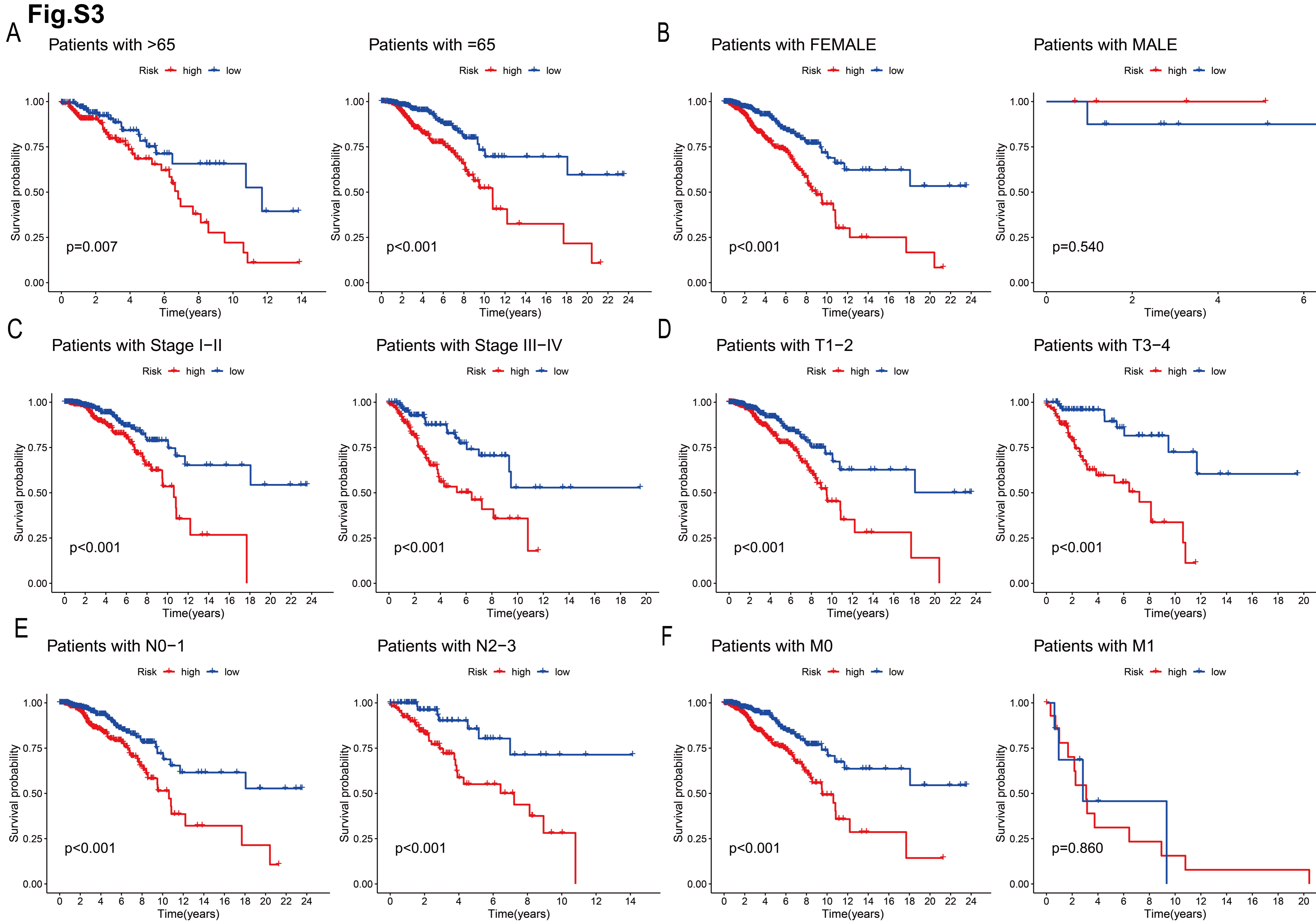

Supplement: Supplementary Figure 3 — Survival curves of different clinical subgroups based onrisk scores. The model was applicable to samples from different clinical groups, primarily showing survival differences in terms of A age (>65 (P = 0.007) and ≤65 (P < 0.001); B sex (female; P < 0.001), C overall stage (P < 0.001), D T stage (P < 0.001), and E N stage (P < 0.05). F The model was not applicable to male patients (P = 0.540) and those with M1 stage tumors (P = 0.860). [file Image3.pdf]

Figure S4

A

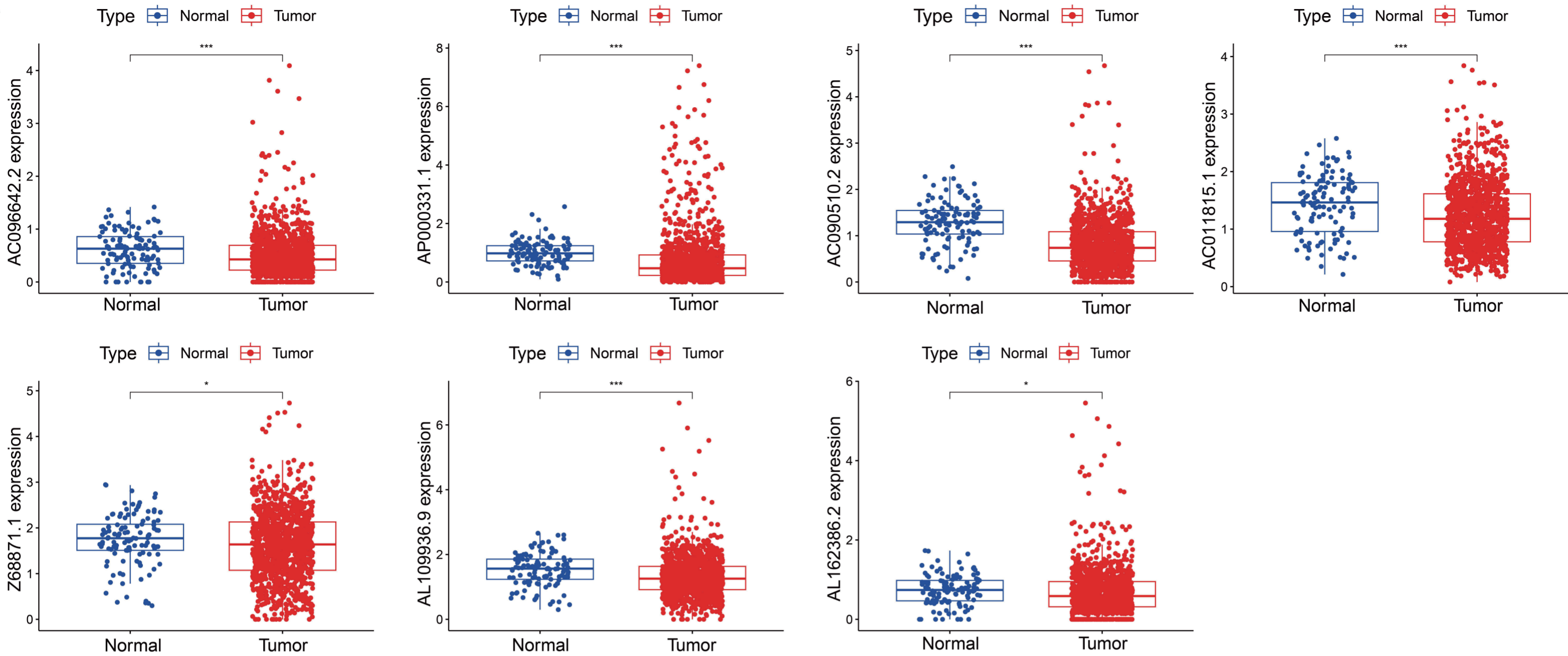

B

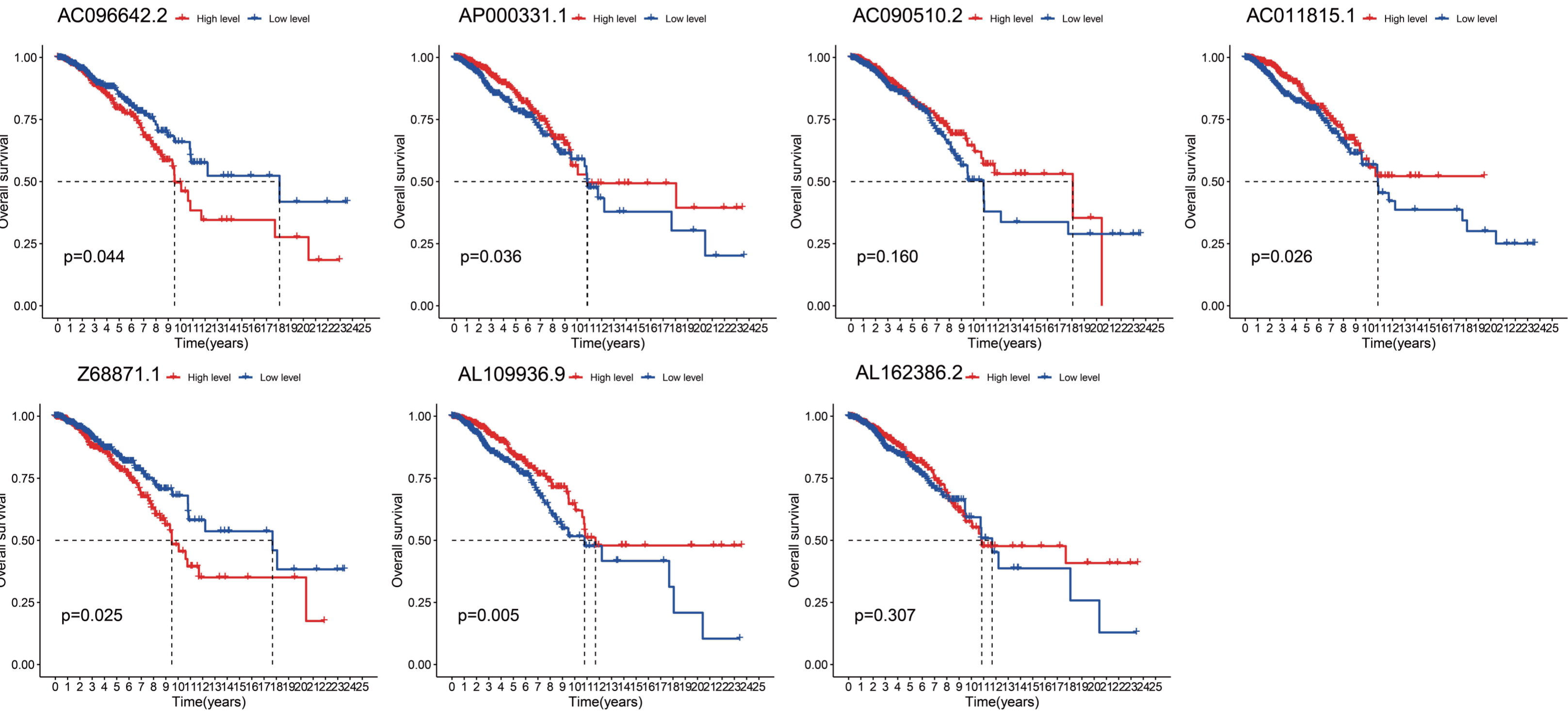

C

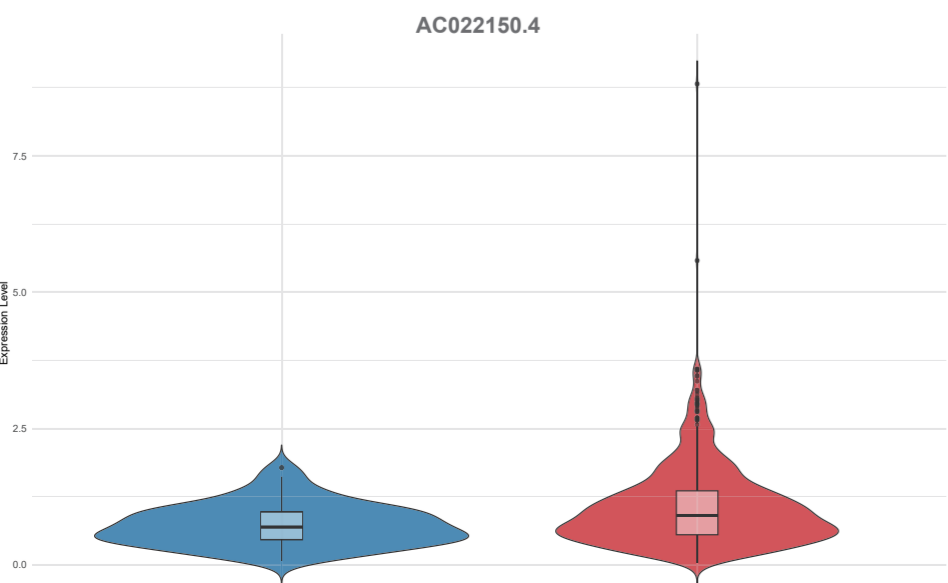

Supplement: Supplementary Figure 4 — Expression and survival plots of the seven lncRNAs used for modeling. A The lncRNAs AC096642.2, AP000331.1, AC090510.2, AC011815.1, Z68871.1, AL109936.9, and AL162386.2 were expressed at low levels. B Low expression levels of AC096642.2 (P = 0.044) and Z68871.1 (P = 0.025) were associated with higher OS. Conversely, high expression levels of AP000331.1 (P = 0.036), AC011815.1 (P = 0.026), and AL109936.9 (P = 0.005) were associated with higher OS, while high or low expression levels of AC090510.2 and AL162386.2 were not associated with significant differences in OS (P > 0.05). C. The external dataset TANRIC confirmed the differential expression of AC022150.4 between tumor and normal cells (P<0.05). *:P < 0.05, **:P<0. 01, ***:P<0. 001. [file Image4.pdf]

Fig.S5

A

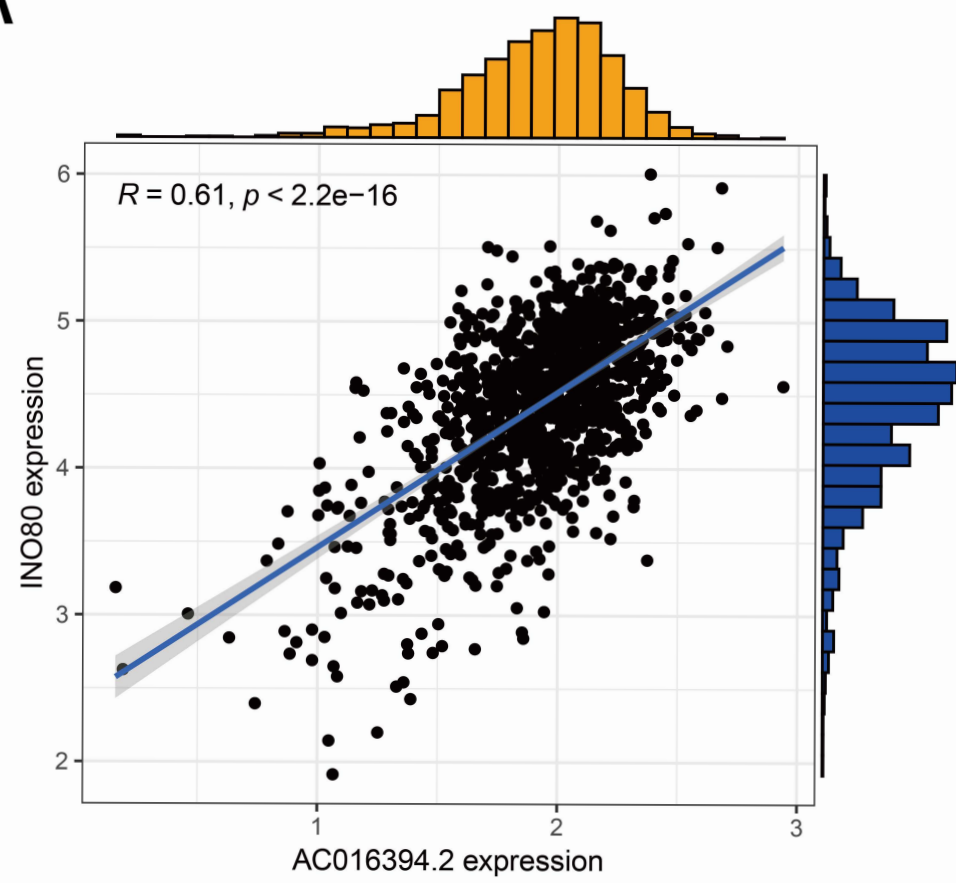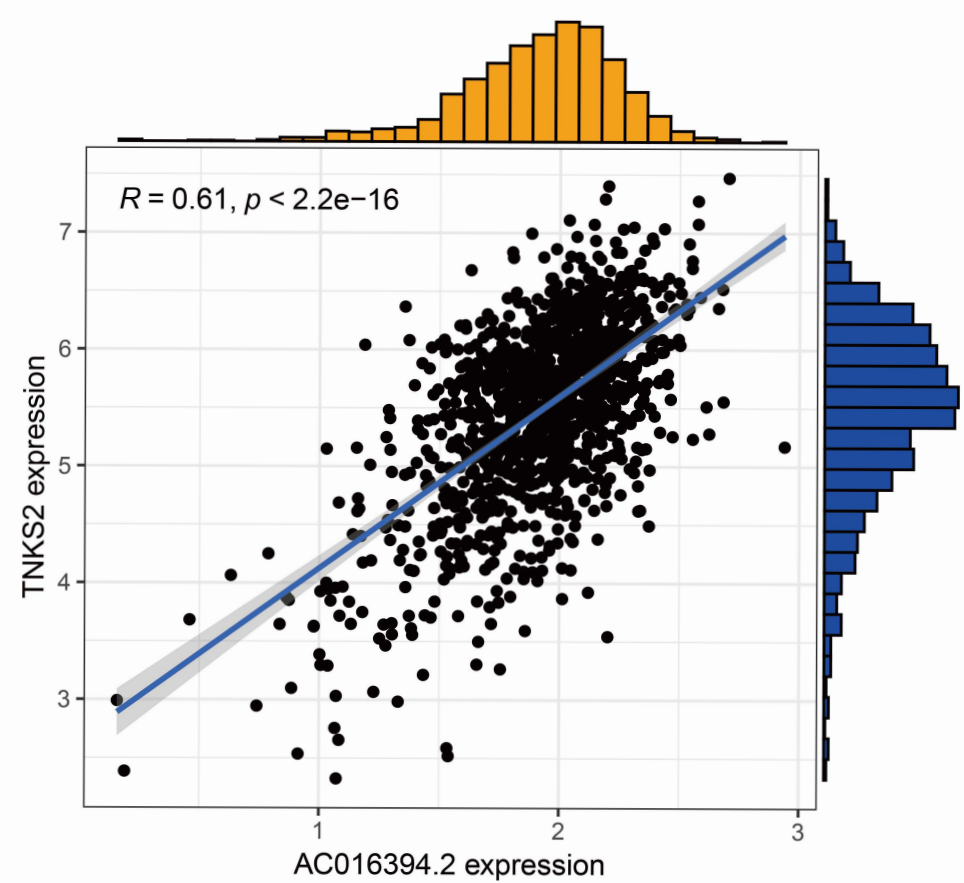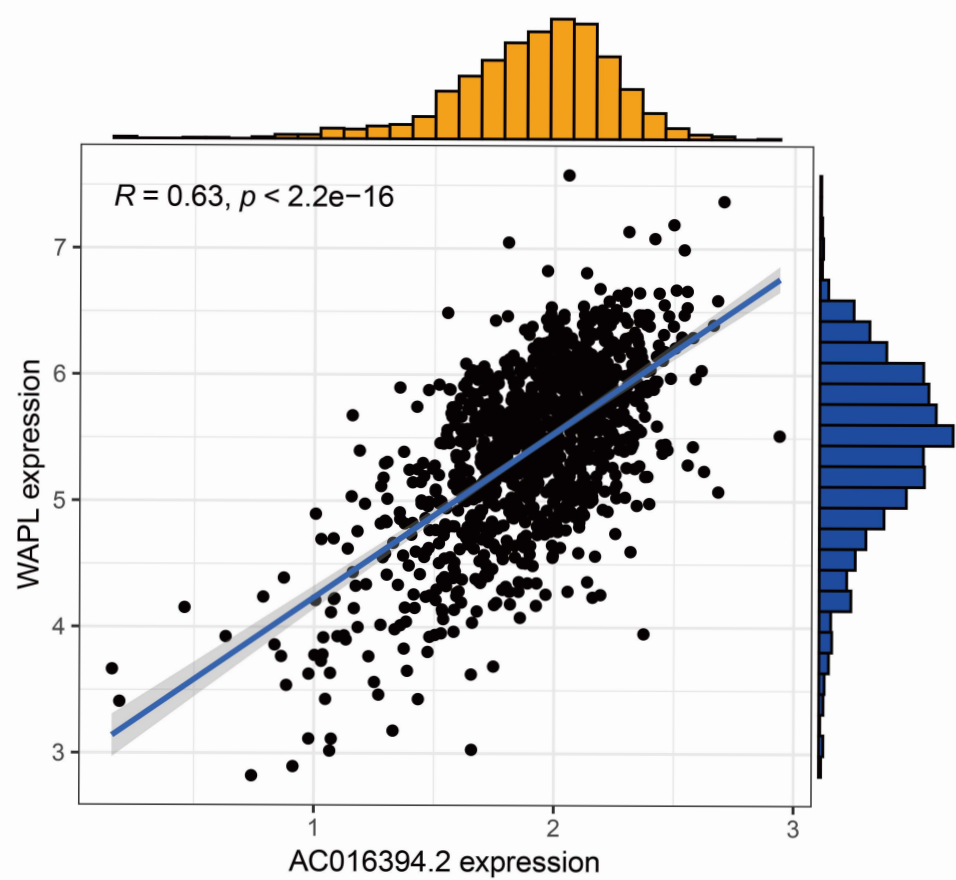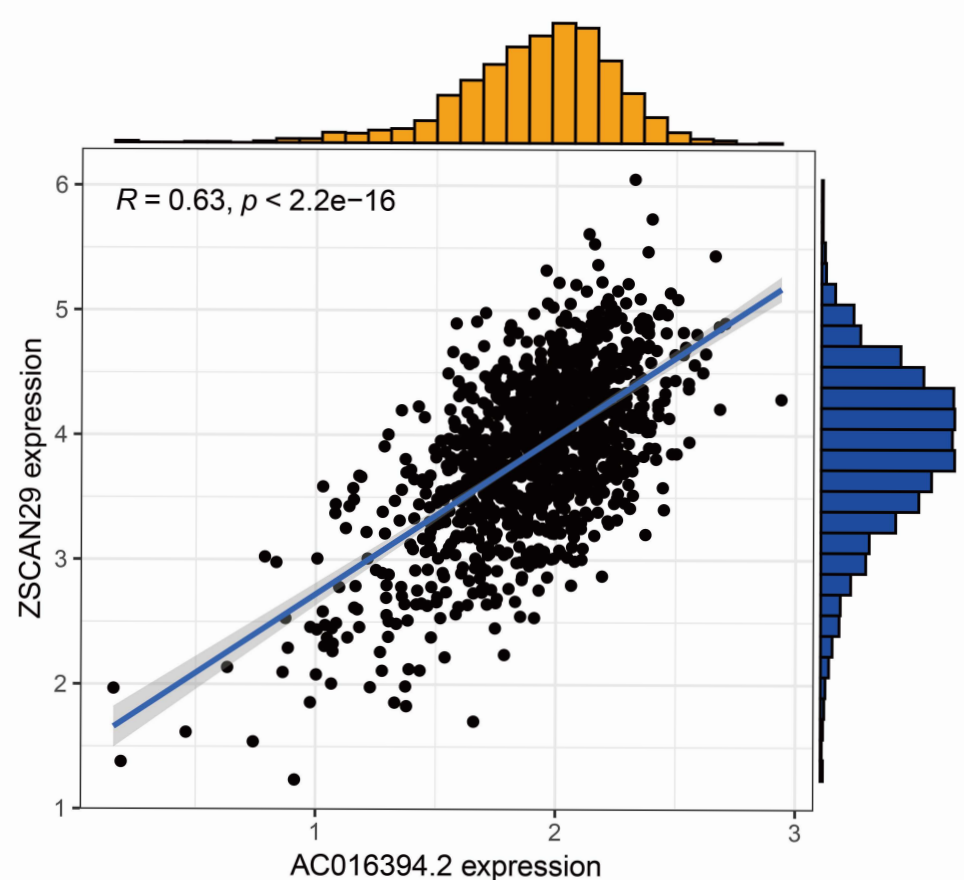

B

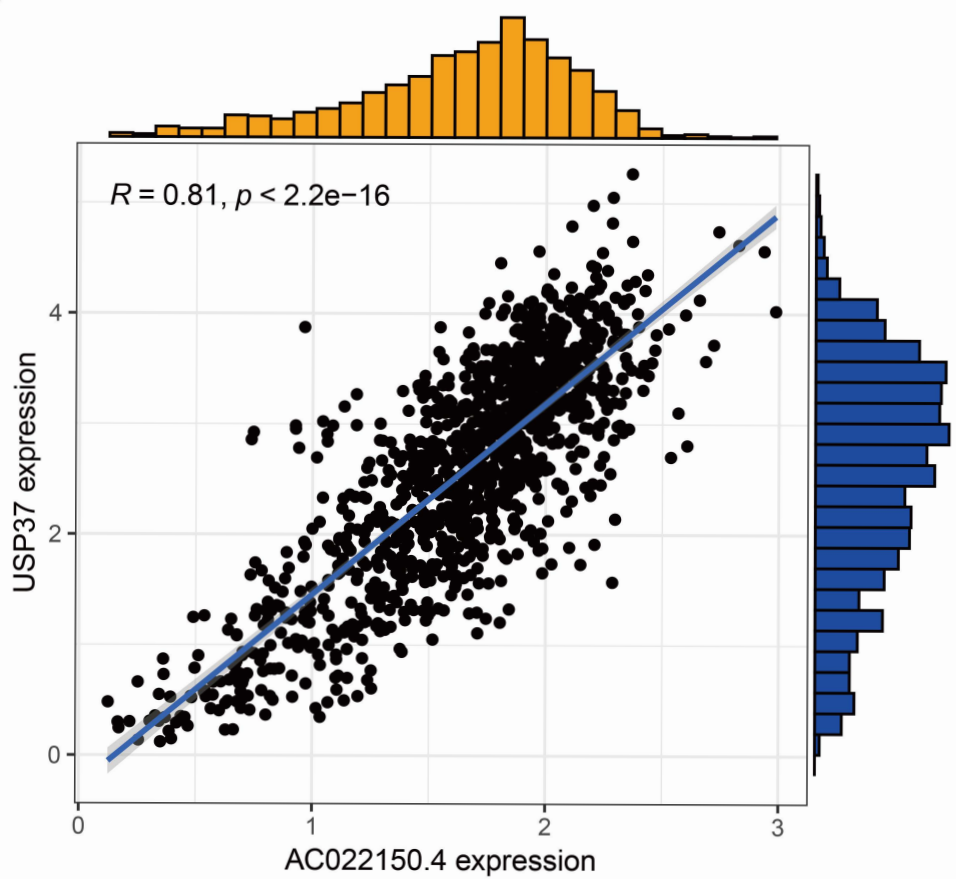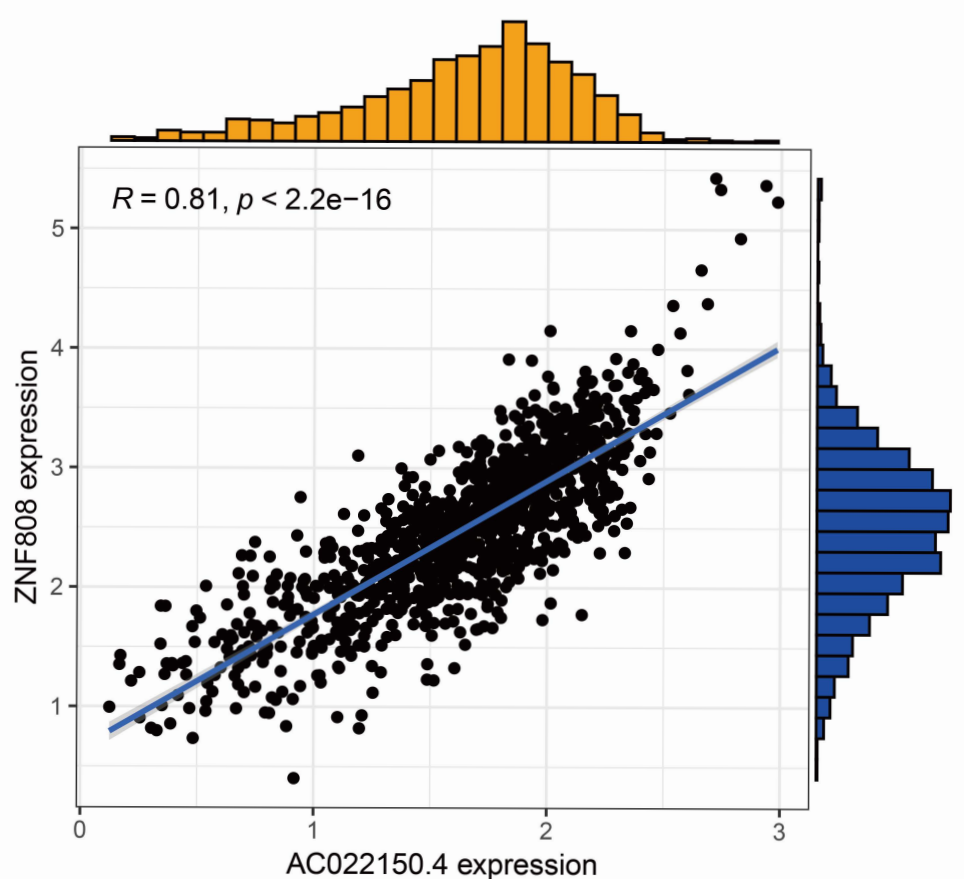

Supplement: Supplementary Figure 5 — Genes co-expressed with AC016394.2 and AC022150.4. A Correlation between AC016394.2 and the co-expressed genes, INO80, TNKS2, WAPL, and ZSCAN29. B Correlation between AC022150.4 and the co-expressed genes, USP37 and ZNF808. [file Image5.pdf]
